# Pandemic responsiveness: Evidence from social distancing and lockdown policy during COVID-19

**DOI:** 10.1371/journal.pone.0267611

**Published:** 2022-05-19

**Authors:** Timothy Besley, Sacha Dray

**Affiliations:** Department of Economics, London School of Economics and Political Science, London, England, United Kingdom; Konkuk University, KOREA, REPUBLIC OF

## Abstract

We study changes in social distancing and government policy in response to local outbreaks during the COVID-19 pandemic. Using aggregated county-level data from approximately 20 million smartphones in the United States, we show that social distancing behaviors have responded to local outbreaks: a 1% increase in new cases (deaths) is associated with a 3% (11%) increase in social distancing intensity. Responsiveness is reinforced by the presence of public measures restricting movements, but remains significant in their absence. Responsiveness is higher in high-income, more educated, or Democrat-leaning counties, and in counties with low health insurance coverage. By contrast, social capital and vulnerability to infection are strongly associated with more social distancing but not with more responsiveness. Our results point to the importance of politics, trust and reciprocity for compliance with social distancing, while material constraints are more critical for being responsive to new risks such as the emergence of variants.

## 1. Introduction

The COVID-19 pandemic has highlighted the need for timely responses to health crises. A range of non-pharmaceutical interventions (NPIs) has been deployed worldwide to curb the progression of the pandemic. Social distancing measures highlight the trade-off between private costs and social benefits of NPIs. Individuals are being asked to sacrifice their self-interest to curb the community-wide spread of the disease by reducing their social contacts. Changes in risks associated with infections—such as the emergence of new variants—will also influence compliance with social distancing. Though governments play a role by regulating behaviors with stay-at-home orders and the closure of public places, private compliance with these measures remain critical. Knowledge of the groups most susceptible to follow health recommendations and to respond to changes in risk is therefore essential for the effectiveness of public health interventions [1, 2].

An extensive literature has emphasized the importance of the trust that citizens place in their government for the effective functioning of government. [3] coined the term “quasi-voluntary compliance” to emphasize that effective states require the support of their citizens. In that sense, compliance is not a mere product of punishment cost but also depends on the perceived legitimacy of the policy objectives. Scholars have studied the importance of consent and compliance in the context of tax evasion [4, 5], conscription [6], organ donation [7] and voter turnout [8]. Social distancing is a case in point to study the interplay of public and private actions, as the effectiveness of public health guidance crucially depends on the timely cooperation of citizens given the limited coercive power of governments.

This paper investigates the determinants of social distancing and its responsiveness to local outbreaks using data from United States counties between January 21, 2020 and January 29, 2021, measuring social distancing as daily changes in time spent at home and contacts with others at commercial venues from approximately 20 million smartphones. We examine the determinants of social distancing, its responsiveness to local outbreaks, and the relationship between social distancing and lockdown decisions.

In line with earlier work ([9–23]), we find that social distancing varies across counties and is associated with health, economic, and political characteristics of counties. In particular, higher levels of social distancing are associated with a higher share of population aged 65 and older, a higher share of population with risk factors for COVID-19, a higher median household income and/or a higher share of Bachelor’s degree. We highlight the importance of two main factors: political division and social capital. Counties with a 1 standard deviation increase in Democrat vote share in 2016 (or, equivalently, a 15 percentage points increase in vote share) had on average a 3 percentage points higher increase in social distancing. A 1 SD increase in social capital index, capturing both trust in institutions and the strength of social networks, is associated with a 1.9 percentage points increase in social distancing, an effect size larger than that of the health, economic, or education characteristics mentioned above. This result highlights the importance of trust when complying with costly measures to protect oneself and others from health risks. Our specification controls for the time path of the pandemic, the number of recent COVID-19 deaths and cases, and include county as well as state × day fixed effects to account for heterogeneous state policy responses. Our results are consistent with contemporaneous studies (described below) and add to the existing evidence by looking at multiple factors influencing compliance with social distancing, quantifying their relative importance, and showing the specific role of social capital.

Our main contribution is to estimate the responsiveness to COVID-19 risk by measuring how much additional social distancing is associated with increased local COVID-19 infections and mortality.

First, we find significant responsiveness to COVID-19 outbreaks. A 1% increase in new cases (deaths) in the last 7 days is associated with a 3% (11%) increase in social distancing intensity in a county. We estimate responsiveness to be more pronounced in the presence of state policy restricting movements (stay-at-home orders or closure of non-essential shops), but muted forms of responsiveness remain in the absence of these measures. In the absence of lockdown, a 1% increase in new cases (deaths) in the last 7 days is associated with a 2.4% (3.5%) increase in social distancing in a county. As a result, public measures can reinforce private responsiveness, but individuals also independently adapt their behaviors to outbreaks.

Second, we find significant determinants of responsiveness that are different from factors associated with higher intensity of social distancing overall. Counties with a higher median income, higher level of education, and a higher share of uninsured appear more responsive. However, while vulnerability and social capital are predictive of higher compliance with social distancing, they are not associated with more responsiveness. Taken together, these findings suggest that economic conditions and the material cost of infections are key factors for adaptive behavioral change during the pandemic. Our contribution is to separately estimate the determinants of social distancing and those of responsiveness to outbreaks.

Third, we focus on the responsiveness of policies targeted at movement restriction which constitutes a direct attempt by governments to induce social distancing behaviors. We find that a 1% increase in new cases (deaths) is associated with a 3% (5%) increase in the probability of imposing movement restriction. We also find suggestive evidence that measures imposing movement restrictions are more responsive to outbreaks in states with a centralized health governance, and with a higher share of vulnerable population.

The remainder of the paper is organized as follows. Section 2 briefly reviews the related literature and highlights the value-added of our contributions. Section 3 discusses data. Section 4 presents the determinants of social distancing. Section 5 explores the responsiveness of social distancing to local outbreaks. Section 6 looks at the responsiveness of government policy on movement restriction. Section 7 summarizes contributions and Section 8 provides a discussion.

## 2. Contribution

This paper contributes to a growing literature, summarized below, on how the pandemic has affected individual and government behavior.

Interest in these issues did not begin with COVID-19 and a range of prior contributions has looked at the determinants of responses to pandemic (see, for example, [1, 2, 24, 25]). It has been argued that effective public responses to an emerging pandemic requires clear communication and trust [1, 26–30]. Behavioral responses to pandemics such as seeking information and complying with public health measures tend to change as the pandemic develops [31, 32] or official guidance changes [33].

We test the hypothesis that material as well as non-material conditions will matter for determining responsiveness to the pandemic. Following [34], we also hypothesize that responsiveness, i.e. changes in social distancing in response to higher infections and deaths, will also respond to non-material factors.

Following the literature, we provide precise estimates of the factors associated with higher compliance with health recommendations and responsiveness to increased risk in the context of social distancing during the COVID-19 pandemic. We highlight the presence of voluntary compliance with health recommendations in the absence of lockdown measures and the increase in private responsiveness during lockdowns.

Our paper adds to existing knowledge in three main ways. First, we focus on *responsiveness* to the evolution of the pandemic. We provide estimates of how changes in social distancing and the likelihood of imposing mobility restrictions are affected by the prevalence of the disease as measured by changes in local cases and deaths. Prior work such as [35] provides evidence that death counts affects perceived risk of contagion and self-reported intention to comply with social distancing by exploiting delays in death reports in Mexico. Similarly, [31] found that higher H1N1 incidence in U.S. states were associated with greater perceived risk of infection, but did not find changes in self-reported compliance with pharmaceutical interventions. [36] found that social distancing increases more in response to cases in counties where individual are historically more willing to incur individual costs to contribute to social objectives, while it decreased in counties with less community engagement. [37] provide evidence that social network exposure to COVID-19 cases shapes individuals’ social distancing behavior using Facebook data in the early months of the pandemic. Our contribution is to estimate public and private responsiveness to local COVID-19 outbreaks measured by deaths and cases. We explore a wide range of factors explaining individual responsiveness to outbreaks including social capital, health, political and economic factors. Additionally, we provide evidence that state policy was similarly responsive to increases in cases and deaths, and that statewide stay-at-home orders increased individual responsiveness to local outbreaks.

Our second contribution is to generalize and augment findings from a range of other recent research on compliance with social distancing during the COVID-19 pandemic [9–14]. In common with this work, we find significant differences in social distancing based on partisan differences [15–17], trust and social capital [10, 18–21], and income [22, 23]. Our contribution is to test for multiple factors associated with social distancing, to explore a wider range of characteristics associated with vulnerability to COVID-19 in addition to age, and to quantify the relative importance of health, socioeconomic and political characteristics for the compliance with social distancing through the use of standardization. Our results stress the importance of political partisanship and social capital for compliance with social distancing in counties before age, health characteristics and income.

Third, we develop a new measure of social distancing in U.S. counties by using location data from a large sample of smartphones from two sources [38, 39]. Compared to other indexes, our measure captures two distinct dimensions of social distancing (i) staying at home and (ii) avoiding others at commercial venues, and is consistent with trends observed in Google mobility reports used by other studies.

## 3. Data

### 3.1. COVID-19 deaths and cases

We use the daily count of COVID-19 cases and deaths compiled by [40] from official state and local sources, covering nearly all U.S. counties since the first reported COVID-19 case on January 21, 2020. S2 Fig in S1 Appendix panels A and B show the geographic variation in deaths and cases from COVID-19 across counties as of January 29, 2021. This highlights the higher severity of the pandemic in the South and Midwest.

### 3.2. Social distancing

We construct a social distancing index based on smartphone location data and relying on two underlying dimensions: (i) changes in mobility (ii) exposure to other phone users. These capture stay-at-home recommendations as well those to avoid crowded places. Our social distancing index is constructed as a weighted average of these two measures arrived at using a principal component analysis (PCA). The S1 Appendix provides details on data construction. We validate this index by comparing it with Google mobility metrics and find very similar national trends, as shown in the S1 Appendix.

### 3.3. Mobility

To measure compliance with stay-at-home orders, we use the Mobility and Engagement index (MEI) developed by [39], who rely on aggregated mobile device data from the company *SafeGraph* on 16 to 20 million smartphones. Their index is a principal component analysis of seven distinct metrics capturing time spent at home. The metrics are (1) fraction of devices leaving home in a day (2) fraction of devices away from home for 3–6 hours at a fixed location (3)fraction of devices away from home longer than 6 hours at a fixed location (4) average daytime hours spent at home (5) fraction of devices taking trips longer than 16 kilometers (6) fraction of devices taking trips less than 2 kilometers (7) average time spent at locations far from home. See [39] for more details. We then express this measure as percentage reduction in time outside home compared to a national average between January 3 and March 1, 2020.

### 3.4. Exposure to others

To measure social contact, we rely on the device exposure index (DEX) developed by [38]. They use daily GPS location from a sample of approximately 20 million devices. DEX quantifies the county-level average of the number of other devices that a given device has been exposed to through visits at commercial venues. This index is a proxy for exposure to COVID-19 through social contact. Commercial venues account for about 750,000 venues such as retail stores, restaurants, convenience stores and bars that are sufficiently small so that visiting devices would be exposed to each other. This excludes the venue categories “Residential”, “Nature and Outdoor”, “Theme Parks”, “Airports”, “Universities”. See [38] for more details. The sample of devices covers the 2,018 counties that were the residential county of at least 1,000 devices on every day from January 6 to 12, 2020, to ensure a sufficiently large device sample for each county. These counties account for 94% of the U.S. population in 2019.

We express exposure as a percentage change compared to a pre-pandemic national baseline in January-February 2020. We use a national baseline following [39]’s construction of the MEI index. Specifically, let *X*_*csdt*_ denote the exposure level in county *c* on date *d* in state *s* after *t* days since the cumulative deaths exceeded 3 and let *P* denote pre-pandemic social contacts level measured as the average exposure weighted by the county-level population, between January 21 and February 28, 2020. Baseline exposure ends on February 28 as February 29 is the first U.S. reported death from COVID-19. We exclude days of higher social contacts during public holidays on January 20 and February 17 to be conservative.

Our core exposure measure is therefore:

$$\begin{array}{c} \rho_{csdt}=100-\left(X_{csdt}/P*100\right). \end{array}$$ (1)

Trends and geographic variations in social distancing index are shown in the S1 Appendix. S1 Fig in S1 Appendix highlights the co-movements between social distancing and infections during the first 5 months of the pandemic, and its attenuation since July 2020 with the relative stability of national social distancing levels despite rises in cases and deaths. S2 Fig in S1 Appendix Panel C and D shows the wide differences in social distancing across the country, and points to greater variation in social distancing over time in the South. S3 Fig in S1 Appendix reports that both dimensions of social distancing used are closely related, and follow similar trends as national reduction in time outdoor measured by the Google mobility reports.

### 3.5. Lockdown policy

We capture the decision to impose a lockdown as a dummy variable for whether a state imposed a statewide stay-at-home order. Information on such orders come from [41]. We also use a measure of business closure to capture less strict policy of movement restriction. This data comes from the Center for the Ecology and Infectious Diseases (CEID at UGA), see https://github.com/CEIDatUGA All but 5 States have imposed a lockdown which started between March 17 and April 7, 2020. The first state to lock down is California. Of those states, all have eased lockdown since then. Easing of lockdown took place initially between April 20 and June 9. On average, this first phase of lockdown lasted for 1.5 months (47 days on average). Most of the policy response to COVID-19 is implemented by States and the Federal government in the United States ([42]). Although there exist some local policy variation within states as pointed by [43], we do not have access to a comprehensive database covering these local policies.

### 3.6. County and state characteristics

Data on county characteristics come from the American Community Surveys (ACS) 2014–18, the USDA, MIT election lab and U.S. Census’s Small Area Health Insurance Estimates Program in 2018. The share of population with at least 3 risk factors associated with COVID-19 comes from the Census Community Resilience Estimate based on data from the 2018 ACS, population estimates and the National Health Interview Survey (NHIS) data. They identify 9 ACS-defined risk factors for households and individuals based on living conditions and socio-economic characteristics, and 3 health conditions risk factors from the NHIS, encompassing a wide array of risk dimensions related to COVID-19. Demographic characteristics are measured in 2010 and come from the USDA ERS. We treat Black, Hispanic and Asian as mutually exclusive categories, so that Black are defined as non-Hispanic African-American.

State characteristics are compiled from the Correlates of State Policy, CDC classification of state health governance. Governor information from the National Governor Association. We use the share of essential workers estimated by the Bureau of Labor Statistics based on classification by CISA.

## 4. Determinants of social distancing

In this section, we describe the main determinants of social distancing in the United States.

### 4.1. Empirical specification

We observe social distancing *ρ* in county *c* on day *d* based on the index of social distancing discussed above. The core specification is the following:

$$\begin{array}{c} \rho_{csdt}=Z_{c}\cdot \beta +\gamma_{sd}+\gamma_{t}+\eta D_{ct}+\varepsilon_{csdt} \end{array}$$ (2)

where *Z*_*c*_ is a vector of standardized economic, health, political and cultural characteristics of a county. Health characteristics are the share of uninsured, the share of population aged 65 years and older, and the share of population with at least 3 risk factors for COVID-19. Economic characteristics are the median household income and the share of population with a bachelor degree. We control for political differences using the share of Democrat vote in 2016. We use votes in the 2016 Presidential elections and not 2020 to reduce concerns that 2020 votes might reflect views on the pandemic and not political preferences. Social Capital is measured using the Social Capital project index from the U.S. Joint Economic Committee constructed in 2018. We use standardized measures to allow for comparability in the magnitudes of the coefficients across variables. We control for the level of COVID-19 infection with *D*_*ct*_ which is the log of new deaths and cases from COVID-19 in county *c* at time *t* using a moving average over the last 7 days. We use a moving average to account for reporting issues that affect the day deaths are counted. All specifications also include county-level demographic controls for the log of population and demographic characteristics (share of Asian, Black and Hispanic). In addition, we add an outbreak time fixed effect *γ*_*t*_ measured as dummies for the relative time since cumulative deaths reached 3 in a county to control for the typical time path of an outbreak. We also add a state × day fixed effects, *γ*_*sd*_, to capture differences in timing of state-level policy e.g. stay-at-home orders that directly influence social distancing. Thus in terms of controls, Eq 2 is quite demanding. The key parameter of interest is $\hat{\beta }$ which quantifies the relationship between county characteristics on social distancing.

Table 1 presents the results. To isolate each factor, we first run our empirical specification with each characteristics of interest separately in Columns 1–7, then move to a more flexible specification with all characteristics included in Column 8. We discuss below results for health, economic, political, and cultural (social capital) characteristics.

**Table 1:** Determinants of social distancing.

	(1)	(2)	(3)	(4)	(5)	(6)	(7)	(8)
	*Dependent variable: Social Distancing Index*							
% Aged 65 Years and Older	2.484***							1.654***
	(0.280)							(0.427)
% with 3+ Risk Factors for COVID-19		1.734***						1.479***
		(0.285)						(0.538)
% without Health Insurance			0.759					0.181
			(0.583)					(0.674)
Median Household Income				0.525				1.517***
				(0.324)				(0.495)
% with Bachelor’s Degree					0.434			-1.375**
					(0.354)			(0.553)
% Vote Democrat 2016						2.507***		2.858***
						(0.496)		(0.543)
Social Capital Index							1.821***	1.911***
							(0.467)	(0.570)
Log Deaths Last 7 days	1.828***	1.966***	2.650***	2.924***	2.908***	2.950***	2.843***	1.760***
	(0.494)	(0.477)	(0.505)	(0.503)	(0.488)	(0.508)	(0.506)	(0.456)
Log Cases Last 7 days	2.011***	1.777***	1.696***	1.674***	1.693***	1.843***	1.709***	2.100***
	(0.182)	(0.184)	(0.187)	(0.184)	(0.186)	(0.189)	(0.185)	(0.179)
Observations	749,832	749,832	749,832	749,832	749,832	747,967	746,103	744,238
Adjusted *R*^2^	0.75	0.74	0.74	0.74	0.74	0.74	0.74	0.75
Mean Dependent Variable	37.6	37.6	37.6	37.6	37.6	37.6	37.6	37.5
State × Day fixed effect	X	X	X	X	X	X	X	X
Outbreak time fixed effect	X	X	X	X	X	X	X	X
Demographic Characteristics	X	X	X	X	X	X	X	X

*Notes*: Standard errors clustered at the county level are in parentheses. Significance levels:

* 10%,

** 5%,

*** 1%.

Each regression is based on Eq 2. The social distancing index measures the average percentage reduction in time outside home and exposure to others at commercial venues compared to January-February 2020. See main text for more details. Unit of observation: county-day. Sample period: January 21, 2020–January 29, 2021. Outbreak time is measured as the time since the first 3 reported COVID-19 deaths in the county. Deaths and cases due to COVID-19 are measured using a moving average over the last 7 days. Demographic characteristics are the log of population and share of Asian, Black and Hispanic. The mean and standard deviation in parenthesis for the unstandardized county characteristics are: % Aged 65 Years and Older: 18.37 (4.55)% with 3+ risk factors for COVID-19: 25.89 (5.05), % without Health Insurance: 11.49 (5.04), Median Household Income: 52,793 (13,884), % with Bachelor’s Degree: 21.57 (9.37), % Vote Democrat 2016: 31.53 (15.23), Social Capital Index: 0.00 (1.00).

#### 4.1.1. Health conditions

We find that variables representing health-related vulnerability to COVID-19 are associated with more social distancing. First, counties with a higher share of older population have higher levels of social distancing. As Column 1 in Table 1 shows, a 1 standard deviation (SD) increase in the share of the population aged 65 years or older (or, equivalently, a 4.55 percentage points increase) is associated with a 2.5 percentage points increase in social distancing. This effect is statistically significant and large in magnitude and robust to controlling for the level of infection through the log of cases and deaths in the last 7 days, as well as confounding factors such as county demographic characteristics. Second, COVID-19 specific risk factors derived from the Census Bureau also predict social distancing. As noted in Section 3, these risk factors assess the vulnerability of population to being infected with the disease based on a multi-dimensional approach encompassing health and general living conditions. These populations are less likely to be resilient and we regard it to be a convincing measure of the share of population with higher vulnerability to COVID-19. We find that a 1 SD increase in share of vulnerable population (this corresponds to a 5 percentage points increase in the share of vulnerable population) increases social distancing significantly by 1.6 percent, even after controlling for other all county characteristics consistent with the idea that health risk factors affect the precautionary behavior of individuals. We find no significant difference in social distancing when looking at the share of population without health insurance, whether separately or when controlling for other factors. Thus there is no evidence that this dimension of material costs is associated with the adoption of social distancing although, as we show below, they could still affect the response to changes in risk.

#### 4.1.2. Economic conditions

As Column 4 shows, we find no significant association between median household income and social distancing. However, income is correlated with other socio-economic conditions such as age and education. After including all these variables together though, we find that counties with a 1 SD higher median household income (or, equivalently, an increase of 13,884 USD per year) increase social distancing by 1.5 percentage points. We find no significant relationship between the share of the population with a bachelor’s degree when we do not control for other potential determinants (see Column 5), and a negative effect when controlling for all candidate determinants including median household income.

#### 4.1.3. Politics

Given the political divide in the attitudes towards COVID-19 in the United States [12, 15], we investigate whether politics is a predictor of social distancing. Column 8 of Table 1 shows that the share of the vote for the Democrat party in 2016 is the predictor most strongly associated with higher levels of social distancing. A 1 SD increase in Democrat vote in 2016 (which corresponds to a 15.23 percentage points increase) is associated with a 2.854 percentage points increase in social distancing, an effect that is significant and robust to the inclusion of other county characteristics. We view this as picking up a correlation between political attitudes and responsiveness to the pandemic. This effect is markedly larger than that of age or vulnerability for explaining compliance with social distancing, and is the most important predictor among candidate determinants of social distancing.

#### 4.1.4. Social capital

In our preferred specification, social capital is the second most important correlate of social distancing. We rely on an index of social capital that encompasses the presence of strong social networks, vibrant civil society and trust in institutions. This index summarizes 10 economic, social and demographic indicators measuring the strength of social networks such as the share of single household, number of nonprofit organizations, presidential election voting rate in 2012 and 2016, the share of mail-back response rate for the Census, or the number of violent crimes ([10] also use this indicator to capture civic capital. In addition, they use voter participation separately and the social capital index from [44]. We replicate our analysis to these measure of civic capital in the S1 Appendix). Counties with higher levels of social capital are found to have significantly higher levels of social distancing. This is suggestive of social norms of reciprocity and trust being important predictor of compliance with social distancing measures.

**Fig 1:**
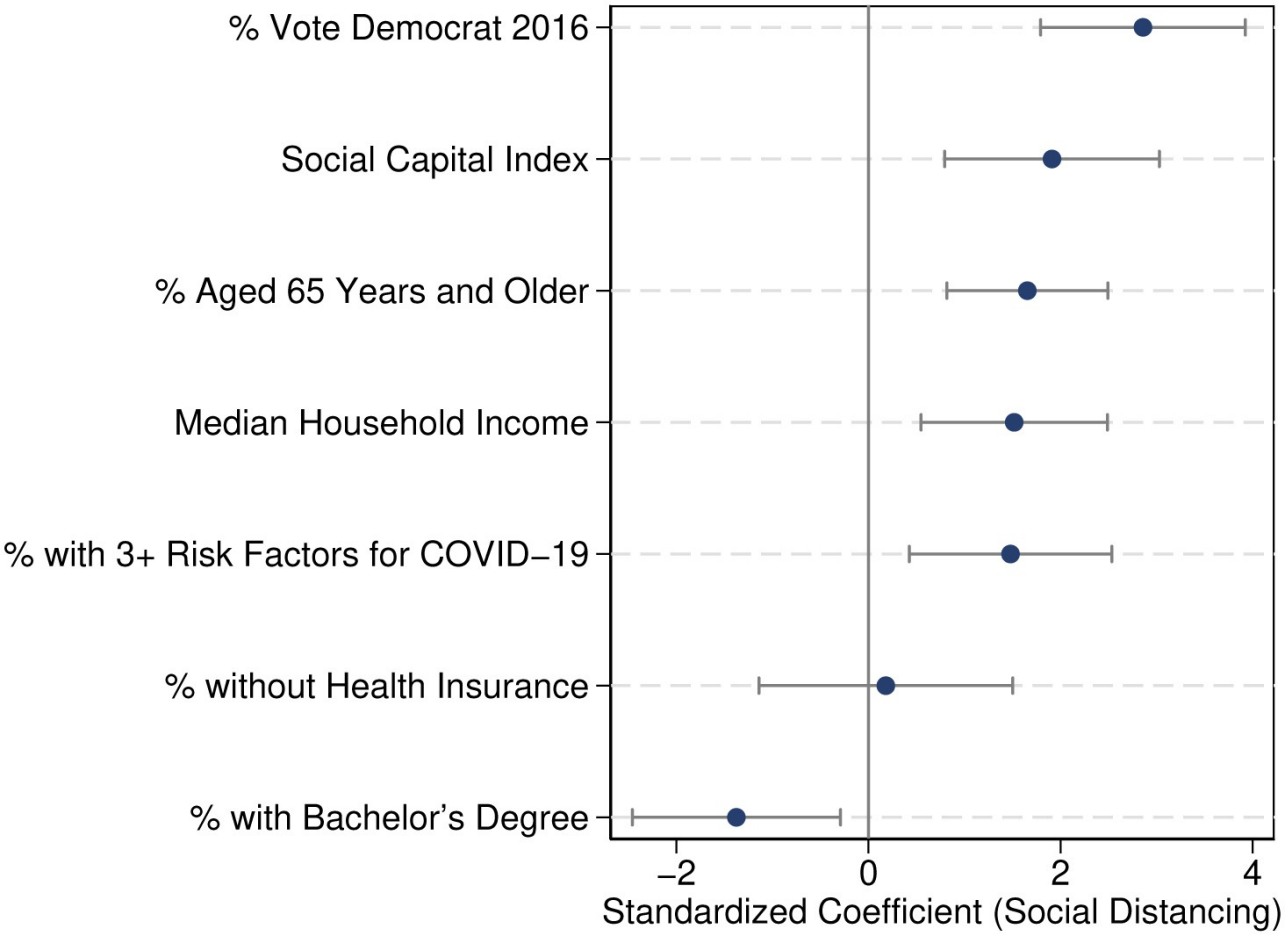
Determinants of social distancing. *Notes*: This figure plots estimates on determinants of social distancing from Table 3. See corresponding footnotes for details.

The results are summarized in Fig 1. Consistent with other evidence, we find that vulnerability to COVID-19 and income are predictors of compliance with social distancing measures. However, political preferences and the level of social capital are more important predictors of compliance. This reflects the importance of the political divide, trust in others and the strength of social community for the success of non-pharmaceutical interventions (In the S1 Appendix, we replicate our findings looking at each of the dimensions of social distancing separately). Our results are consistent with recent evidence for earlier periods of the pandemic (e.g. [10]), and provide complementary findings controlling for several dimensions of COVID-19 risks in addition to age. We also contribute to the literature by quantifying the relative importance of health, economic and political factors and find stronger associations between levels of social distancing and social capital or political partisanship than with economic or health factors.

## 5. Responsiveness to local outbreaks

Our main contribution measures how compliance with social distancing evolved with the progression of the disease. From a conceptual point of view, individuals make assessments based on the best available information to assess risks, in particular information from local outbreaks. We would therefore expect social distancing to be greater in counties where the outbreak is more severe. Policy can matter for voluntary compliance if the presence of stay-at-home orders affects citizens’ belief about risk.

Existing studies have tended to focus on the determinants of social distancing, as in the previous section, but not drivers of responsiveness. Our results generate novel insights by looking at how individuals increase compliance with social distancing when the number of cases or deaths increase in their county. Throughout, we explore responsiveness to either COVID-19 cases or deaths as the information on risk could have been conveyed by either indicator and almost certainly change during the pandemic. This is a key element of responsiveness, and intuitively captures changes in behaviors in response to greater risk which makes sense as citizens will have pretty good access to data on how the disease is evolving locally, and should perceived a greater risk when reported cases or deaths are rising ([31, 35]). As well as looking at average responsiveness, we will also explore heterogeneous effects, i.e. how responsiveness varies with county characteristics.

### 5.1. Empirical specification

We observe the intensity of social distancing *ρ* in county *c* on day *t* based on the measure of social exposure discussed above. The core specification is the following:

$$\begin{array}{c} \rho_{csdt}=\gamma_{c}+\gamma_{sd}+\gamma_{t}+\eta D_{ct}+\varepsilon_{csdt} \end{array}$$ (3)

where *D*_*ct*_ is the log of new deaths from COVID-19 in county *c* on day *t* using a moving average over the last 7 days. We use a moving average to account for reporting issues that affect the day deaths are counted. We also take the log of 1 + new deaths to avoid observations with 0 new deaths being dropped. We include county fixed effects *γ*_*c*_ to control for fixed differences in exposure between locations, and an outbreak time fixed effect *γ*_*t*_ measured as dummies for the relative time since cumulative deaths reached 3 in a county to control for the typical time path of an outbreak. We also add a state × day fixed effects, *γ*_*sd*_, to capture differences in the timing of state-level policy e.g. stay-at-home orders that directly influence social distancing. The key parameter of interest is $\hat{\eta }$ which measures changes in social distancing in response to the progression of the disease. This parameter captures how current social distancing decisions are affected by past local outbreaks. To the extent that social distancing behaviors are a response to anticipated future outbreaks, we assume that expectations expectations about future outbreaks are derived from past infections and deaths, and we therefore interpret this coefficient as responsiveness to past outbreaks.

### 5.2. Citizen responsiveness

The results are reported in Table 2 and show a positive link between COVID-19 deaths and social distancing, with highly robust point estimates. In Panel A, we focus on responsiveness to new deaths over the last 7 days using a moving average. We find that a 1% increase in new reported COVID-19 deaths is associated with a 10 percentage points increase in social distancing, a significant and large effect. Part of this response may be driven by policy changes in movement restrictions. In Columns 2 and 3, we interact infection rates with policy restricting movement (lockdown and business closure respectively) to measure both overall responsiveness and policy-driven responsiveness. As reported in Column 2, we find that citizens were more than twice as responsive during stay-at-home orders compared to when these orders are not in place. A similar pattern can be found when looking at policy shutting down non-essential shops as shown in Column 3. We also find that individuals are also responsive to cases and deaths in the absence of lockdown. This suggests that individual responsiveness is not entirely driven by policy decisions, but also comes from other behavioral changes.

**Table 2:** Citizen responsiveness to COVID-19 outbreaks.

	(1)	(2)	(3)
	*Dependent variable: Social Distancing Index*		
**A: Responsiveness to New Deaths**			
Log Deaths Last 7 days	10.64***	3.533***	4.633***
	(0.397)	(0.309)	(0.466)
Log Deaths Last 7 days × Lockdown		4.903***	
		(0.520)	
Log Deaths Last 7 days × Non-essential shops closed			3.878***
			(0.539)
Observations	750,713	750,340	601,487
Adjusted *R*^2^	0.36	0.88	0.87
Mean Dependent Variable	37.6	37.6	37.4
**B: Responsiveness to New Cases**			
Log Cases Last 7 days	2.936***	2.452***	2.320***
	(0.182)	(0.169)	(0.192)
Log Cases Last 7 days × Lockdown		2.747***	
		(0.183)	
Log Cases Last 7 days × Non-essential shops closed			2.911***
			(0.212)
Observations	749,957	749,957	601,143
Adjusted *R*^2^	0.88	0.88	0.88
Mean Dependent Variable	37.6	37.6	37.4
**C: Responsiveness to Cumulative Deaths**			
Log Cumulative Deaths	2.602***	2.232***	2.344***
	(0.215)	(0.201)	(0.236)
Log Cumulative Deaths × Lockdown		2.679***	
		(0.166)	
Log Cumulative Deaths × Non-essential shops closed			2.435***
			(0.178)
Observations	750,465	750,465	601,577
Adjusted *R*^2^	0.88	0.88	0.88
Mean Dependent Variable	37.6	37.6	37.4
**D: Responsiveness to Cumulative Cases**			
Log Cumulative Cases	3.894***	3.358***	3.308***
	(0.219)	(0.212)	(0.232)
Log Cumulative Cases × Lockdown		1.732***	
		(0.113)	
Log Cumulative Cases × Non-essential shops closed			1.907***
			(0.134)
Observations	750,465	750,465	601,577
Adjusted *R*^2^	0.88	0.88	0.88
Mean Dependent Variable	37.6	37.6	37.4
County fixed effect	X	X	X
State × Day fixed effect	X	X	X
Outbreak time fixed effect	X	X	X

*Notes*: Standard errors clustered at the county level are in parentheses. Significance levels:

* 10%,

** 5%,

*** 1%.

Each regression is based on Eq 3 and includes county, state × day and outbreak time fixed effects. The social distancing index measures the average percentage reduction in time outside home and exposure to others at commercial venues compared to January-February 2020. See main text for more details. Unit of observation: county-day. Sample period: January 21, 2020–January 29, 2021. Outbreak time is measured as the time since the first 3 reported COVID-19 deaths in the county. Deaths and cases due to COVID-19 over the last 7 days are measured using a moving average.

Overall, we show that compliance with social distancing increases when either cases or deaths in the county, and is robust to alternative counting of death or case reporting. A possible concern in interpreting the findings is is that responsiveness may be sensitive to the precise measure of infection being used. In Panels B-D we replicate this analysis using cumulative instead of new counts, and cases instead of deaths. The results for these variants are qualitatively similar. In the S1 Appendix, we also replicate our findings looking at each of the dimensions of social distancing separately, and we finds similar results when looking at responsiveness to either cases or deaths over the last 14 or 21 days. This reinforces the findings that individuals have been generally responsive to the severity of the pandemic when complying with social distancing. Further, responsiveness is greater in the presence of policy restricting mobility, which underscores the complementarity between policy and private action.

### 5.3. Determinants of responsiveness

We now explore determinants of responsiveness to local outbreaks. To do so, we run the following regression:

$$\begin{array}{c} \rho_{csdt}=\gamma_{c}+\gamma_{sd}+\gamma_{t}+\eta d_{ct}+\psi \left(d_{ct}\times Z_{c}\right)+\theta \left(d_{ct}\times X_{c}\right)+\varepsilon_{csdt} \end{array}$$ (4)

where we interact the log of new COVID-19 deaths with standardized county characteristics *Z*_*c*_ that we have shown can explain social distancing decisions as well demographic characteristics *X*_*c*_. Demographic characteristics are population density, the share of Hispanics, Blacks, and Asians in 2010. As before, we control for county, state, day, state × day and outbreak time fixed effects. The coefficient of interest is $\hat{\psi }$. We explore health, economic and political factors associated with higher responsiveness to local outbreaks in Table 3.

**Table 3:** Determinants of responsiveness to COVID-19 outbreaks.

	(1)	(2)	(3)	(4)	(5)	(6)	(7)	(8)
	*Dependent variable: Social Distancing Index*							
**A: Responsiveness to New Deaths**								
Log Deaths Last 7 days	2.269***	2.433***	2.590***	1.760***	1.309***	1.874***	2.909***	0.742***
	(0.250)	(0.250)	(0.272)	(0.230)	(0.244)	(0.270)	(0.259)	(0.278)
Log Deaths Last 7 days ×% Aged 65 Years and Older	-0.810***							-0.470
	(0.268)							(0.362)
Log Deaths Last 7 days ×% with 3+ Risk Factors for COVID-19		-0.951***						0.243
		(0.365)						(0.539)
Log Deaths Last 7 days ×% without Health Insurance			0.185					1.480***
			(0.403)					(0.437)
Log Deaths Last 7 days × Median Household Income				1.803***				1.503***
				(0.212)				(0.393)
Log Deaths Last 7 days ×% with Bachelor’s Degree					2.221***			1.322***
					(0.250)			(0.377)
Log Deaths Last 7 days ×% Vote Democrat 2016						1.712***		1.048**
						(0.313)		(0.427)
Log Deaths Last 7 days × Social Capital Index							1.364**	-0.924
							(0.551)	(0.609)
Observations	750,340	750,340	750,340	750,340	750,340	748,475	746,610	744,745
Adjusted *R*^2^	0.88	0.88	0.88	0.88	0.88	0.88	0.88	0.88
Mean Dependent Variable	37.6	37.6	37.6	37.6	37.6	37.6	37.6	37.5
**B: Responsiveness to New Cases**								
Log Cases Last 7 days	2.026***	2.053***	2.087***	1.929***	1.731***	1.909***	2.248***	1.621***
	(0.140)	(0.146)	(0.145)	(0.132)	(0.131)	(0.137)	(0.139)	(0.122)
Log Cases Last 7 days ×% Aged 65 Years and Older	-0.169**							-0.0948
	(0.0709)							(0.106)
Log Cases Last 7 days ×% with 3+ Risk Factors for COVID-19		-0.241***						0.168
		(0.0842)						(0.174)
Log Cases Last 7 days ×% without Health Insurance			-0.250					0.245
			(0.153)					(0.174)
Log Cases Last 7 days × Median Household Income				0.540***				0.407***
				(0.0764)				(0.142)
Log Cases Last 7 days ×% with Bachelor’s Degree					0.731***			0.404***
					(0.0749)			(0.117)
Log Cases Last 7 days ×% Vote Democrat 2016						0.919***		0.591***
						(0.0986)		(0.131)
Log Cases Last 7 days × Social Capital Index							0.458***	-0.130
							(0.134)	(0.153)
Observations	749,957	749,957	749,957	749,957	749,957	748,092	746,228	744,363
Adjusted *R*^2^	0.88	0.88	0.88	0.88	0.88	0.88	0.88	0.88
Mean Dependent Variable	37.6	37.6	37.6	37.6	37.6	37.6	37.6	37.5
County fixed effect	X	X	X	X	X	X	X	X
State × Day fixed effect	X	X	X	X	X	X	X	X
Outbreak time fixed effect	X	X	X	X	X	X	X	X
Interaction with Demographic Characteristics	X	X	X	X	X	X	X	X

*Notes*: Standard errors clustered at the state level are in parentheses. Significance levels:

* 10%,

** 5%,

*** 1%.

Each regression is based on Eq 4 and includes county, state × day and outbreak time fixed effects. The social distancing index measures the average percentage reduction in time outside home and exposure to others at commercial venues compared to January-February 2020. See main text for more details. Unit of observation: county-day. Sample period: January 21, 2020–January 29, 2021. Outbreak time is measured as the time since the first 3 reported COVID-19 deaths in the county. Deaths and cases due to COVID-19 are measured using a moving average over the last 7 days.

#### 5.3.1. Health conditions

Contrary to its prediction on *levels* of social distancing, we find no impact of health characteristics on social distancing *responsiveness* to local outbreaks. As reported on Column 8, neither the share of 65 and older or the share of population with 3 or more risk factors to COVID-19 are significantly associated with a higher responsiveness to local outbreaks (a similar result can be found when looking at responsiveness to cases instead of deaths as shown in Panel B). This is consistent with vulnerable populations socially distancing more regardless of changes in infection rates. On the contrary, counties with a higher share of individuals without health insurance are more responsive to local outbreaks, while this did not predict levels of social distancing. Here, we find that a 1 SD increase in the share of uninsured (i.e. a 5 percentage points increase) is associated with a 1.48 percentage points increase in social distancing when COVID-19 deaths increase by 1%. We find a similar effect for responsiveness to cases, although not significant at the 5% level. This is suggestive of material risks of being infecting being important for behavioral change in the face of greater risk, and more generally underscore the importance of looking at the determinants of compliance and responsiveness separately.

#### 5.3.2. Economic conditions

We find that economically advantaged populations are more responsive to outbreaks. Both median household income and the share of those with a bachelor’s degree are positively associated with responsiveness to outbreaks. We find similar results when we look at responsiveness to cases, albeit of a smaller magnitude.

#### 5.3.3. Politics

Political division is found to be an important predictor of responsiveness. The Democrat party vote share in 2016 is associated with more responsiveness to outbreaks. A 1 SD increase in Democrat vote is associated with a 1% increase in responsiveness to new deaths and 0.6% increase in responsiveness to new cases. This mirrors our results on the determinants of social distancing and reinforces the importance that political views on the pandemic can have in shaping the behavior of citizens.

#### 5.3.4. Social capital

We find no robust evidence of social capital being important for responsiveness. We find a positive correlation in a regression without including the other variables discussed above (see Column 7), but this disappears once we add all of the dependent variables together. We similarly find no robust evidence of social capital increasing responsiveness when using alternative measures of social capital used in [10] such as voter participation and the social capital measure developed by [44].

**Fig 2:**
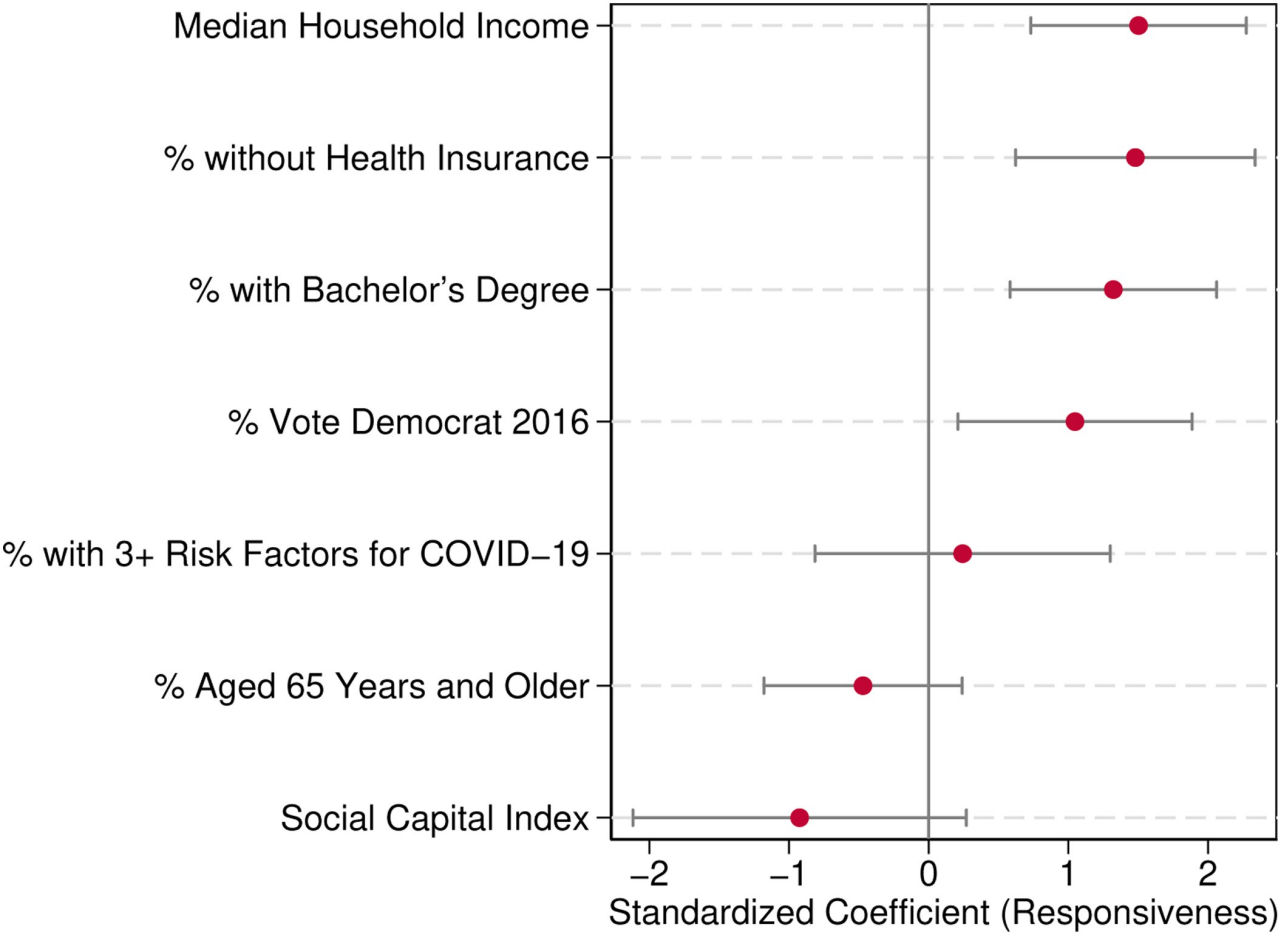
Determinants of responsiveness to COVID-19. *Notes*: This figure plots estimates of responsiveness to new deaths from Table 3. See corresponding footnotes for details.

Our results are summarized in Fig 2. This figure highlights the importance of economic factors and the material cost of being infected for responsiveness to the pandemic, and the absence of significant association between responsiveness and vulnerability or social capital. We also explore the robustness of our results by looking at the responsiveness to cases instead of deaths. We also provide robustness to our results by using only Exposure to Others or Time Outside Home as measure of social distancing, as reported in the S1 Appendix. Individuals may have initially been responding to COVID-19 deaths when deciding to restrict contacts, but they could have shifted their attention to cases as U.S. testing capacities improved. However, we find that this is not the case, as shown in Panel B of Table 3. Another potential concern with our findings is reverse causality. However, we think that, even if this were an issue, it is likely to lead to underestimation of the responsiveness to local outbreaks. To the extent that social distancing is reducing the level of infection, this would underestimate the positive association between infections and social distancing we documented.

## 6. Government responsiveness

Above we explored how citizens respond to local outbreaks. But similar considerations affect how government responds either because they reflect their citizens’ concerns or because they also use the death and case rates to assess risk directly. Either way, we expect policy also to follow the evolution of the pandemic.

The empirical specification that we use to explores this is:

$$\begin{array}{c} MvtRest_{sdt}=\gamma_{s}+\gamma_{d}+\gamma_{t}+\eta d_{sdt}+\varepsilon_{sdt} \end{array}$$ (5)

where *MvtRest* indicate the presence of policy restricting social movements (stay-at-home orders of business closure). The analysis is conducted at the state-level.

The results are reported in Table 4 which shows that policy has been responsive to the pandemic. In Panel A, we look at how changes in COVID-19 deaths are associated with the probability of enforcing a lockdown. Lockdown measures appear the most responsive to increase in deaths or cases. A 1% increase in either deaths or cases in the last 7 days is associated with a 5 percentage points increase in probability of imposing a lockdown. We find similar results looking at cases or deaths over the last 14 or 21 days, as reported in the S1 Appendix. More nuanced measures, such as imposing that non-essential shops be closed, limiting gathering in public space, or a [0, 6] index of non-pharmaceutical intervention, appear more responsive to the cumulative count of cases than new infections or deaths. This is consistent with the most costly measure responding to greater severity of the infection as captured by the extent of mortality, while gradual measures are evolving together with the stock of infected to regulate the path of the disease. In the S1 Appendix, we explore determinants of state policy responsiveness. We find that Pro-Trump governors were less likely to be responsive to COVID-19 deaths when imposing a lockdown, while states with a centralized public health governance and more vulnerable citizens were more responsive. We find no evidence of economic factors being important for state policy responsiveness. Unlike other studies (e.g. [13]), these results provide evidence that both individuals and governments exhibited responsiveness to outbreaks in their decisions regarding social distancing, and reinforce the complementary nature of private and public actions discussed in section 5.

**Table 4:** State government responsiveness to COVID-19.

	(1)	(2)	(3)	(4)
	Lockdown	Non-Essential Shops Closed	Limit Gathering in Public Space	NPI Index
**A: Responsiveness to New Deaths**				
Log Deaths Last 7 Days	0.0521***	0.0332	0.0679*	0.141***
	(0.0104)	(0.0215)	(0.0350)	(0.0401)
Observations	19,615	15,250	19,341	15,250
Adjusted *R*^2^	0.71	0.73	0.53	0.87
Mean Dependent Variable	0.11	0.21	0.53	1.91
**B: Responsiveness to New Cases**				
Log Cases Last 7 Days	0.0282**	0.00824	0.0459	0.0688*
	(0.0111)	(0.0186)	(0.0323)	(0.0368)
Observations	19,623	15,255	19,347	15,255
Adjusted *R*^2^	0.71	0.73	0.52	0.87
**C: Responsiveness to Cumulative Deaths**				
Log Cumulative Deaths	0.0511***	0.0504***	0.0173	0.154***
	(0.0122)	(0.0167)	(0.0342)	(0.0412)
Observations	19,630	15,262	19,356	15,262
Adjusted *R*^2^	0.71	0.73	0.52	0.87
**D: Responsiveness to Cumulative Cases**				
Log Cumulative Cases	0.0526***	0.0307***	0.0764*	0.139***
	(0.0133)	(0.0109)	(0.0424)	(0.0413)
Observations	19,630	15,262	19,356	15,262
Adjusted *R*^2^	0.71	0.73	0.53	0.87
State fixed effect	X	X	X	X
Day fixed effect	X	X	X	X
Outbreak time fixed effect	X	X	X	X

*Notes*: Standard errors clustered at the state level are in parentheses. Significance levels:

* 10%,

** 5%,

*** 1%.

Each regression is based on Eq 5, and includes state, day and outbreak time fixed effects. Unit of observation: state-day. Outbreak time is measured as the time since the first 3 reported COVID-19 deaths in the county. Deaths and cases due to COVID-19 are measured using a moving average over the last 7 days. Lockdown is a dummy variable indicating that a curfew or lockdown are in place for at least part of the day. The NPI index measures the intensity of movement restriction policy on a scale of 0 to 6. See main text for details.

## 7. Summary and limitations

### 7.1. Summary

This paper makes two main contributions. First, it develops a novel social distancing measure to replicate and extend some existing findings on how social distancing has evolved during the pandemic. In line with other studies, the results show that vulnerability, economic circumstances, social capital and politics are robustly correlated with social distancing behaviors. We interpret these as reflecting material costs and benefits of social distancing as well as less tangible motives related to trust and belief in the importance of pro-social actions which appear to be important factors for compliance. Our results also underscore that political division and social capital are important correlates of compliance relative to economic and health circumstances.

Second, the paper has explored determinants of citizen and government responsiveness to the COVID-19 pandemic. Both public and private actions do appear responsive to cases and deaths. Moreover, we have shown evidence that public and private responsiveness are complements, with citizens responding more to deaths when the government has imposed restrictions on mobility. Economic conditions and the material cost of infection are the important determinants of responsiveness rather than social capital and having a high-risk population. This finding highlights the importance of material constraints for behavioral change when the risk of infection changes, for example with the emergence of new variants.

### 7.2. Limitations

Certain limitations should be born in mind in interpreting the results. First, the associations that we have uncovered between social distancing behaviors and county characteristics, are not sufficient to establish a causal link between these characteristics and social distancing. Second, the study is focused exclusively on social distancing in U.S. counties during the period of January 2020–January 2021, since this is the period for which we have data on mobility changes. However, responsiveness could well be different in other settings. In particular, as shown in S1 Fig in S1 Appendix, social distancing has been declining after the initial lockdown period. The emergence of new variants and changes in the infection prevalence or underlying risk might also affect determinants of social distancing behavior in future. Comparing patterns of responsiveness in other countries, the role of trust in governments in how citizens respond to the pandemic, and the evolution of responsiveness over time, are fruitful areas for future research.

## 8. Discussion

The COVID-19 pandemic has led to renewed emphasis on measures such as social distancing that rely on pro-social acts in addition to having private benefits. To garner compliance, governments can act in conventional ways by regulating behaviors e.g. closing bars and sports venues or limiting how businesses can operate. But there is also a major role for public persuasion and messaging to encourage a sense of social responsibility and promote compliance.

Our results highlight the importance of social cohesion and non-material conditions for the success of compliance with health measures. We show that the level of social distancing is highly correlated with political and social considerations. In contrast, responsiveness to changes in risk is associated with material conditions such as income and health insurance coverage. This suggests that changes to social distancing following new risks– for example with the emergence of variants–will tend to be affected by material constraints, while politics and social considerations are more predictive of overall levels of compliance. On COVID-19 variants, see for instance [45–47].

There is still much to be learned about how citizens and government have behaved in the unprecedented circumstances induced by the COVID-19 pandemic. However, the robust findings uncovered here from a novel source of data provide some important pointers that will help to guide future work as well providing insights into the patterns of responsiveness that we have observed so far.

## Supporting information

S1 Appendix Supporting material: Data construction for the social distancing index, appendix figures and tables. (PDF)

S1 Data (ZIP)

S2 Data (ZIP)

S3 Data (ZIP)

S4 Data (ZIP)

S5 Data (ZIP)

S6 Data (ZIP)

S7 Data (ZIP)

S8 Data (ZIP)

S9 Data (ZIP)
