# Supplementary material for: Pandemic responsiveness: Evidence from social distancing and lockdown policy during COVID-19
Source: PLoS One. 2022 May 19;17(5):e0267611. doi: 10.1371/journal.pone.0267611 (PMC9119550; doi:10.1371/journal.pone.0267611)
Supplement: S1 Appendix — (PDF) [file pone.0267611.s001.pdf]

# Supporting Information: "Pandemic responsiveness: Evidence from social distancing and lockdown policy during COVID-19"

Timothy Besley* Sacha Dray†

April 21, 2022

## Contents

1 Data Construction for the Social Distancing Index 1

2 Appendix Figures and Tables 2

*London School of Economics t.besley@lse.ac.uk
†London School of Economics s.s.dray@lse.ac.uk

## List of Figures


S1 Trends in Social Distancing and COVID-19 Infections . . . . . . . . . . . . . . . 2
S2 Geographic variation in COVID-19 Severity and Social Distancing . . . . . 3
S3 Trends in Social Distancing . . . . . . . . . . . . . . . . . . . . . . . . . . . . . . 4
S4 Geographic Differences in Social Distancing Measures: SDIST vs MEI . . . 5


## List of Tables


S1 Determinants of Social Distancing: Exposure to Others . . . . . . . . . . . . . 6
S2 Determinants of Social Distancing: Time Outside Home . . . . . . . . . . . . 7
S3 Citizen Responsiveness To COVID-19: Exposure to Others . . . . . . . . . . . 8
S4 Citizen Responsiveness To COVID-19: Time Outside Home . . . . . . . . . . 9
S5 Robustness on Responsiveness To COVID-19 . . . . . . . . . . . . . . . . . . . 10
S6 Determinants of Responsiveness To COVID-19 Outbreaks: Exposure to Others . . . . . . . . . . . . . . . . . . . . . . . . . . . . . . . . . . . . . . . 11
S7 Determinants of Responsiveness To COVID-19 outbreaks: Time Outside Home . . . . . . . . . . . . . . . . . . . . . . . . . . . . . . . . . . . . . . . 12
S8 Determinants of Social Distancing: Alternative Social Capital Measures . . 13
S9 Determinants of Responsiveness To COVID-19 outbreaks: Alternative Social Capital Measures . . . . . . . . . . . . . . . . . . . . . . . . . . . . . . 14
S10 Determinants of State Government Responsiveness To COVID-19 . . . . . 15
S11 Robustness on State Government Responsiveness To COVID-19 . . . . . . 16

# 1. Data Construction for the Social Distancing Index

This section describes the construction of the social distancing index by combining aggregated smartphone location data at the county-level from the Mobility and Engagement index (MEI) and the Device Exposure index (DEX). Both data sources provide anonymised and aggregated information on mobility changes, and are publicly available only.

**Mobility and Engagement index (MEI)** The Dallas Fed MEI summarizes the information in seven different variables based on geolocation data collected from a large sample of mobile devices. The data is originally the Social Distancing Metric database from *SafeGraph* but the aggregate county-level database used in this study is publicly available at https://www.dallasfed.org/research/mei. MEI measures the deviation from normal mobility behaviors induced by COVID-19. Details on construction and methodology of the raw dataset can be found in Atkinson et al. (2020). We use the daily scaled county dataset. The data was last accessed on March 17, 2021.

**Device Exposure index (DEX)** The DEX dataset is an one of the exposure indices derived from *PlaceIQ* movement data by Couture et al. (2020). The data is publicly available at https://github.com/COVIDExposureIndices/COVIDExposureIndices. We use the county-level dex (device exposure) dataset that measures the average number of distinct devices that visited the same commercial venues visited by a device as a proxy for social exposure. The data was last accessed on March 17, 2021.

We adapt the MEI and DEX datasets to construct the social distancing index. These data construction steps are necessary to express both underlying indexes in terms of social distancing intensity, and to ensure their comparability. The corresponding data construction steps are the following:

1. We take the negative values of MEI to express the magnitude of mobility reduction.
2. We descale the MEI index so that it no longer has a value of 100 for the week starting April 5, but gives the raw percentage deviation in mobility reduction compared to Jan 3 and Mar 1, 2020. Specifically, we measure the average mobility reduction for the week starting in April from the daily county-level data, and obtain a descaling factor of .7362688.
3. We express the DEX as a percentage deviation in exposure compared to national baseline exposure between January 21 and February 28, 2020. Specifically, we apply the

same methodology as in MEI detailed in Atkinson et al. (2020). We measure the baseline exposure as the average DEX value weighted by county-level population. We obtain a baseline exposure value of $P = 205.3828368$ and express the new variable in deviation term using $\rho_{csdt} = 100 - (X_{csdt}/P * 100)$.

4. We take the principal component analysis to form an index of both DEX and MEI. The resulting variable is our social distancing index.

# 2. Appendix Figures and Tables

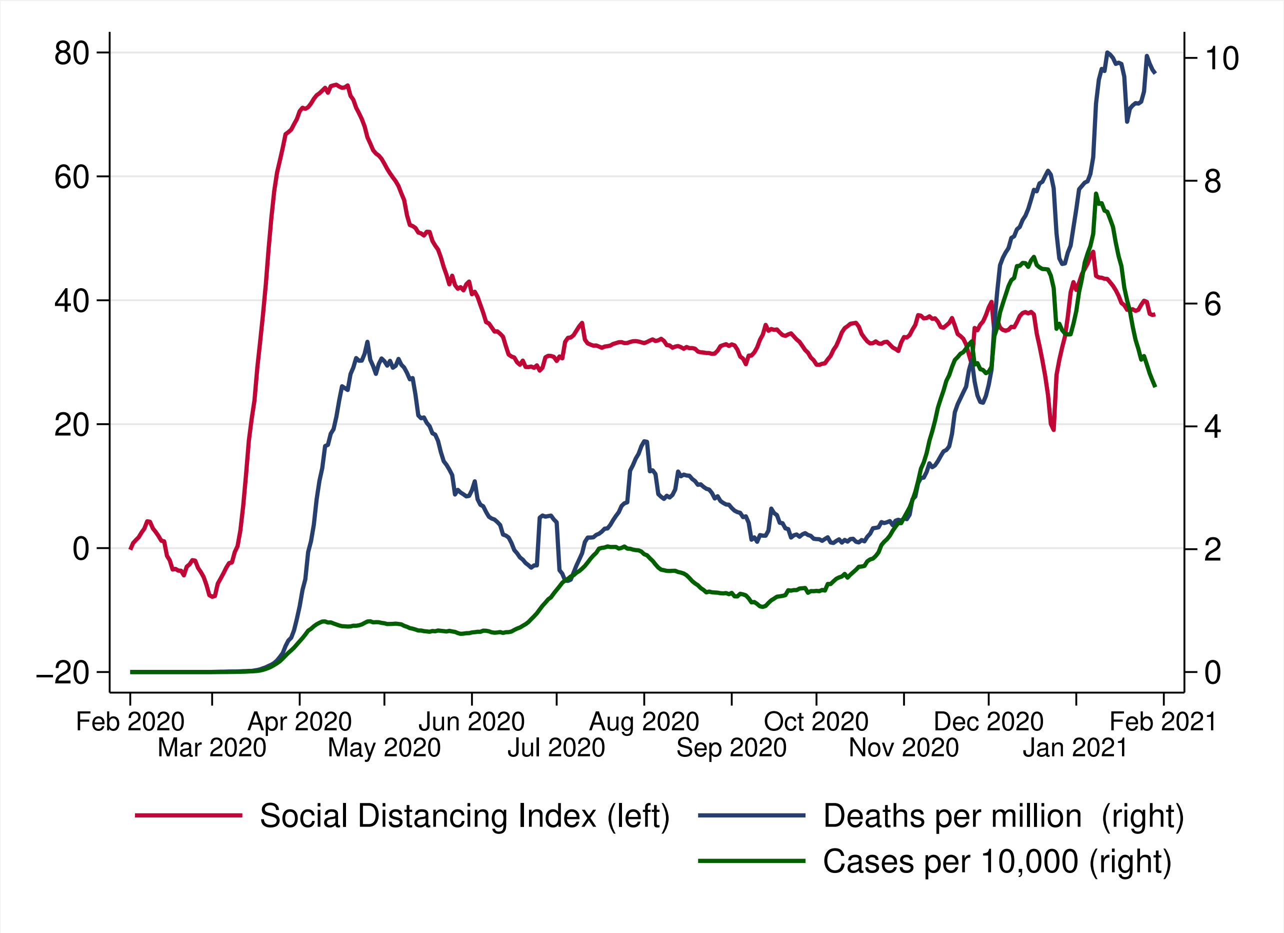


FIGURE S1: **Trends in Social Distancing and COVID-19 Infections**

*Notes:* This figure shows trends in social distancing compared to new COVID-19 deaths and cases per million using a 7-day moving average weighted by county population. The social distancing index is constructed as the average of the percentage reduction in time outside home and percentage reduction in exposure to others at commercial venues compared to January-February 2020. See main text for details.

FIGURE S2: **Geographic variation in COVID-19 Severity and Social Distancing**

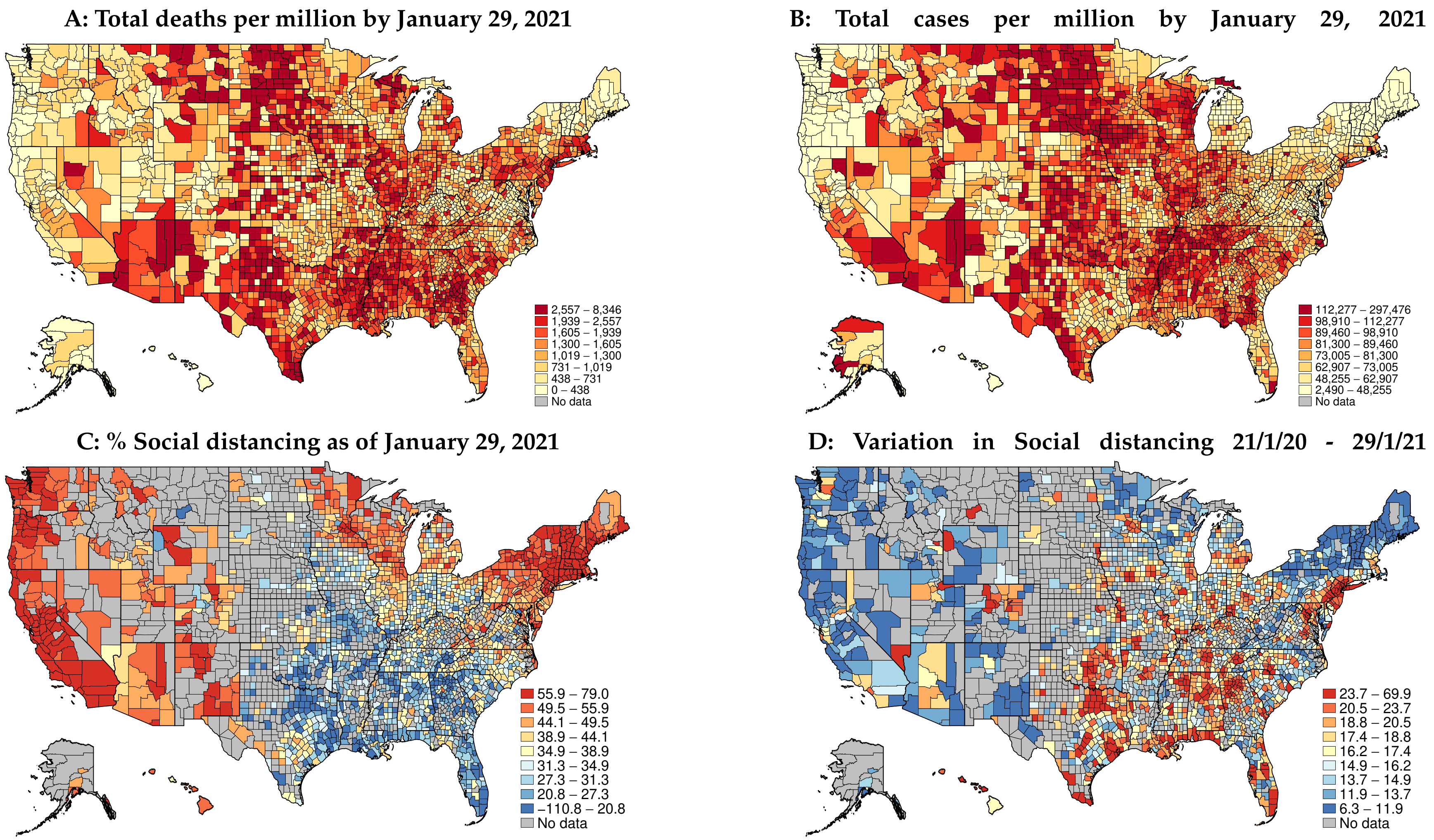


*Notes:* These maps show the geographical variation in COVID-19 infections and social distancing as of January 29, 2021. The social distancing index is constructed as the average of the percentage reduction in time outside home and percentage reduction in exposure to others at commercial venues compared to January-February 2020. Panel D shows the standard deviation in social distancing index between January 21 and January 29, 2021. The social distancing index is measured for the 2,018 counties that were the residential county of at least 1,000 sampled devices on every day from January 6 to 12, 2020, to ensure a sufficiently large device sample for each county. These 2,018 counties account for 94% of the U.S. population in 2019. Maps are created using the maptile command on Stata, which is in the public domain.

**A: Social Distancing Index**

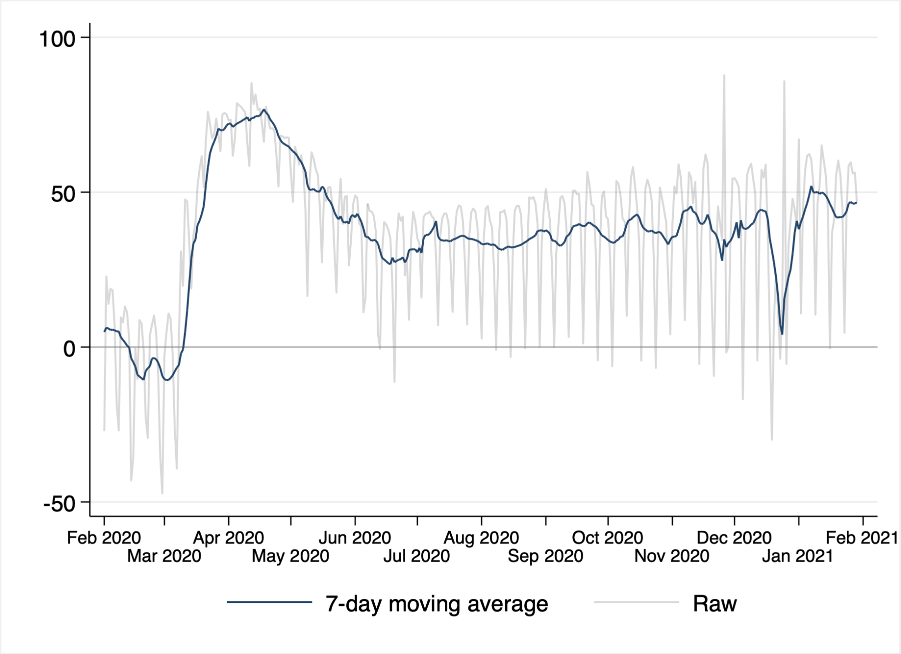


**B: Comparison between Social Distancing Measures**

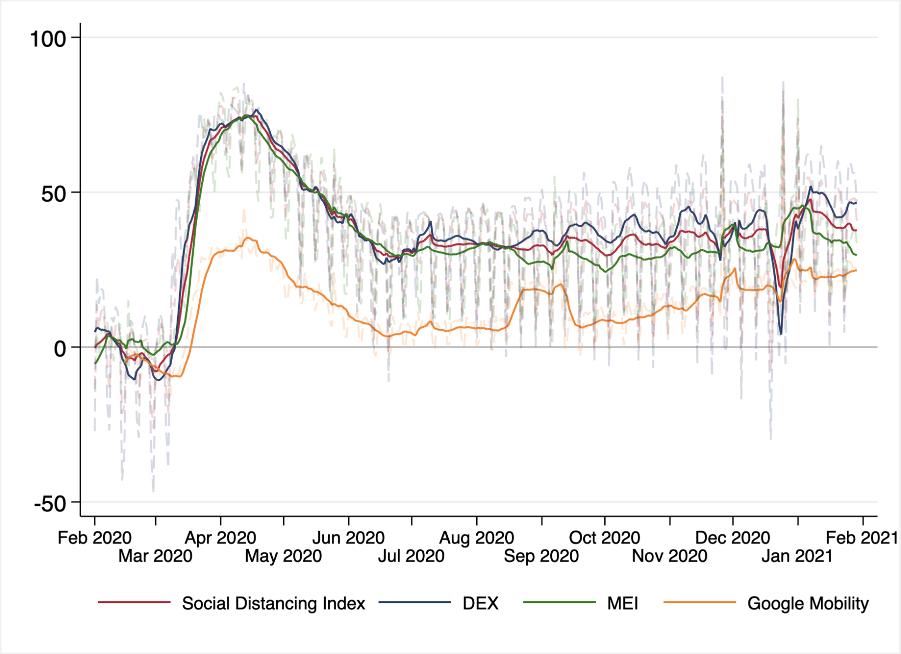


FIGURE S3: **Trends in Social Distancing**

*Notes:* This figures show the raw trends and 7-day moving average of the social distancing index and its two sub-indices weighted by county population. The social distancing index is constructed as the average of the percentage reduction in time outside home (Mobility and Engagement index MEI) and percentage reduction in exposure to others at commercial venues (Device Exposure index DEX) compared to January-February 2020 using a principal component analysis. Google mobility is a measure of the percentage reduction in the number of visitors and time spent in outside places compared to the median value from the 5-week period Jan 3 – Feb 6, 2020. See main text for details.

FIGURE S4: **Geographic Differences in Social Distancing Measures: SDIST vs MEI**

**A: % Social distancing (DEX) as of January 29, 2021**

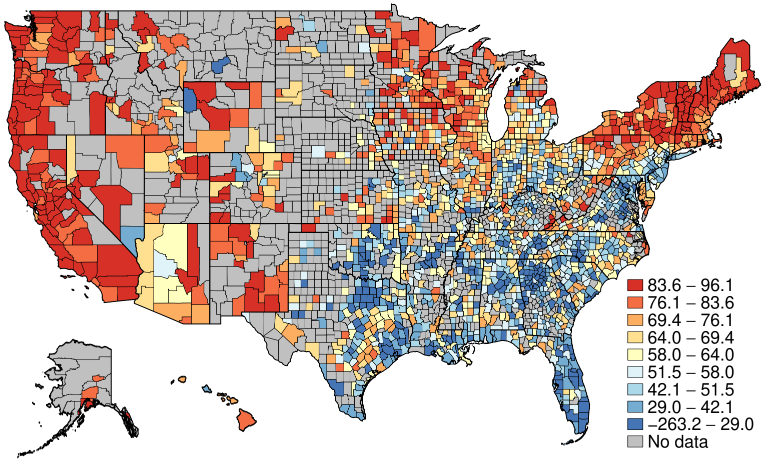


**B: % Social distancing (MEI) as of January 29, 2021**

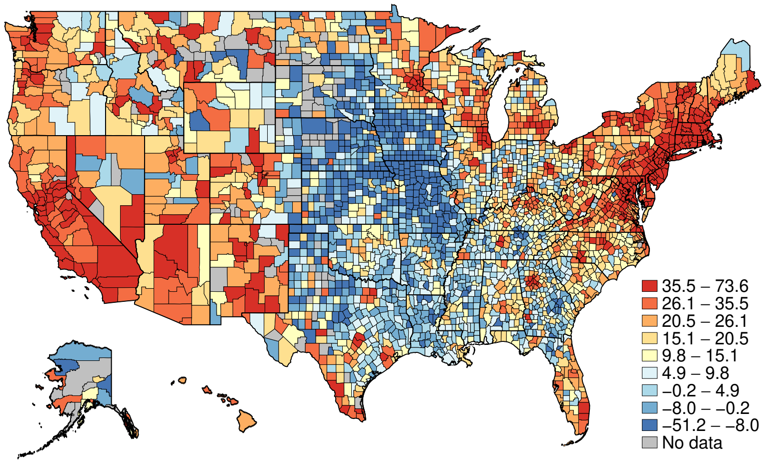


**C: Variation in Social distancing (DEX) 21/1/20 - 29/1/21**

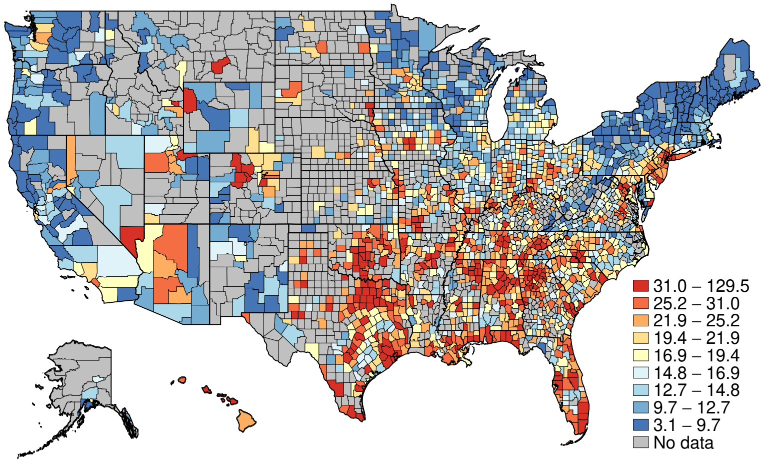


**D: Variation in Social distancing (MEI) 21/1/20 - 29/1/21**

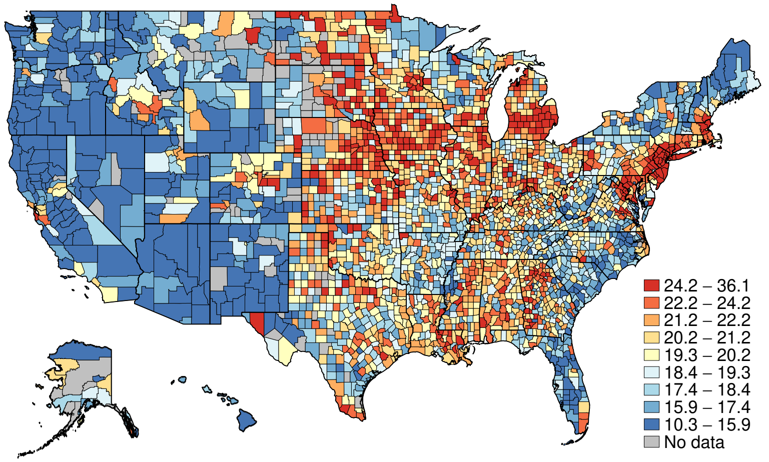


*Notes:* These maps show the geographical variation in social distancing for the two sub-indices of social distancing as of January 29, 2021. The social distancing (DEX) measures the percentage reduction in exposure to others compared to January-February 2020. Social distancing (MEI) measures the percentage reduction in time outside home compared to January-February 2020. Our main measure of social distancing, the social distancing index, combines these indices using a principal component analysis. Panels C and D shows the standard deviation in social distancing between January 21 and January 29, 2021. DEX is measured for the 2,018 counties that were the residential county of at least 1,000 sampled devices on every day from January 6 to 12, 2020, to ensure a sufficiently large device sample for each county. These 2,018 counties account for 94% of the U.S. population in 2019. Maps are created using the maptile command on Stata, which is in the public domain.

## TABLE S1: **Determinants of Social Distancing: Exposure to Others**

| | (1) | (2) | (3) | (4) | (5) | (6) | (7) | (8) |
|---|---|---|---|---|---|---|---|---|
| | | | | *Dependent variable: Social Distancing (DEX)* | | | | |
| % Aged 65 Years and Older | 5.697*** | | | | | | | 3.076*** |
| | (0.555) | | | | | | | (0.833) |
| % with 3+ Risk Factors for COVID-19 | | 6.375*** | | | | | | 2.905*** |
| | | (0.556) | | | | | | (1.061) |
| % without Health Insurance | | | 6.903*** | | | | | 2.105 |
| | | | (1.199) | | | | | (1.325) |
| Median Household Income | | | | -3.882*** | | | | -0.872 |
| | | | | (0.637) | | | | (0.963) |
| % with Bachelor's Degree | | | | | -3.191*** | | | -3.181*** |
| | | | | | (0.670) | | | (1.073) |
| % Vote Democrat 2016 | | | | | | 1.382 | | 3.089*** |
| | | | | | | (0.964) | | (1.093) |
| Social Capital Index | | | | | | | -1.782* | 2.829** |
| | | | | | | | (0.930) | (1.135) |
| Log Deaths Last 7 days | 4.125*** | 3.351*** | 5.289*** | 5.010*** | 5.139*** | 6.371*** | 6.187*** | 2.489*** |
| | (0.993) | (0.963) | (1.006) | (1.010) | (0.974) | (1.031) | (1.031) | (0.911) |
| Log Cases Last 7 days | 2.124*** | 1.703*** | 1.409*** | 1.555*** | 1.414*** | 1.478*** | 1.404*** | 2.172*** |
| | (0.339) | (0.339) | (0.348) | (0.345) | (0.350) | (0.355) | (0.351) | (0.335) |
| Observations | 753,854 | 753,854 | 753,854 | 753,854 | 753,854 | 751,979 | 750,105 | 748,230 |
| Adjusted $R^2$ | 0.63 | 0.63 | 0.62 | 0.62 | 0.62 | 0.62 | 0.62 | 0.63 |
| Mean Dependent Variable | 50.3 | 50.3 | 50.3 | 50.3 | 50.3 | 50.3 | 50.3 | 50.2 |
| State $\times$ Day fixed effect | X | X | X | X | X | X | X | X |
| Outbreak time fixed effect | X | X | X | X | X | X | X | X |
| Demographic Characteristics | X | X | X | X | X | X | X | X |

*Notes:* Standard errors clustered at the county level are in parentheses. Significance levels: * 10%, ** 5%, *** 1%. Social distancing (DEX) measures the percentage reduction in exposure to others compared to January-February 2020.. See main text for more details. Unit of observation: county-day. Sample period: January 21, 2020 - January 29, 2021. Outbreak time is measured as the time since the first 3 reported COVID-19 deaths in the county. Deaths and cases due to COVID-19 are measured using a moving average over the last 7 days.

## TABLE S2: **Determinants of Social Distancing: Time Outside Home**

| | (1) | (2) | (3) | (4) | (5) | (6) | (7) | (8) |
|---|---|---|---|---|---|---|---|---|
| | | | | *Dependent variable: Social Distancing (MEI)* | | | | |
| % Aged 65 Years and Older | -0.513*** | | | | | | | 0.0609 |
| | (0.146) | | | | | | | (0.170) |
| % with 3+ Risk Factors for COVID-19 | | -1.985*** | | | | | | 0.241 |
| | | (0.154) | | | | | | (0.192) |
| % without Health Insurance | | | -3.987*** | | | | | -1.438*** |
| | | | (0.280) | | | | | (0.248) |
| Median Household Income | | | | 4.388*** | | | | 3.983*** |
| | | | | (0.139) | | | | (0.197) |
| % with Bachelor's Degree | | | | | 3.532*** | | | 0.352* |
| | | | | | (0.168) | | | (0.181) |
| % Vote Democrat 2016 | | | | | | 3.023*** | | 2.616*** |
| | | | | | | (0.254) | | (0.241) |
| Social Capital Index | | | | | | | 3.263*** | 0.282 |
| | | | | | | | (0.240) | (0.221) |
| Log Deaths Last 7 days | -0.443 | 0.292 | -0.0932 | 0.621** | 0.249 | -0.451 | -0.801*** | 0.832*** |
| | (0.321) | (0.312) | (0.309) | (0.250) | (0.266) | (0.308) | (0.305) | (0.220) |
| Log Cases Last 7 days | 1.974*** | 1.940*** | 2.036*** | 1.908*** | 1.995*** | 2.217*** | 1.951*** | 2.070*** |
| | (0.106) | (0.104) | (0.102) | (0.0915) | (0.0934) | (0.0990) | (0.101) | (0.0796) |
| Observations | 1,123,566 | 1,123,566 | 1,123,566 | 1,123,566 | 1,123,566 | 1,117,598 | 1,091,173 | 1,085,578 |
| Adjusted $R^2$ | 0.80 | 0.80 | 0.80 | 0.81 | 0.81 | 0.80 | 0.81 | 0.83 |
| Mean Dependent Variable | 22.4 | 22.4 | 22.4 | 22.4 | 22.4 | 22.4 | 22.5 | 22.5 |
| State $\times$ Day fixed effect | X | X | X | X | X | X | X | X |
| Outbreak time fixed effect | X | X | X | X | X | X | X | X |
| Demographic Characteristics | X | X | X | X | X | X | X | X |

*Notes:* Standard errors clustered at the county level are in parentheses. Significance levels: * 10%, ** 5%, *** 1%. Social distancing (MEI) measures the percentage reduction in time outside home compared to January-February 2020. See main text for more details. Unit of observation: county-day. Sample period: January 21, 2020 - January 29, 2021. Outbreak time is measured as the time since the first 3 reported COVID-19 deaths in the county. Deaths and cases due to COVID-19 are measured using a moving average over the last 7 days.

TABLE S3: **Citizen Responsiveness To COVID-19: Exposure to Others**

| | (1) | (2) | (3) |
|---|---|---|---|
| | *Dependent variable: Social Distancing (DEX)* | | |
| **A: Responsiveness to New Deaths** | | | |
| Log Deaths Last 7 days | 9.904*** | 4.463*** | 5.870*** |
| | (0.642) | (0.568) | (0.873) |
| Log Deaths Last 7 days × Lockdown | | 8.185*** | |
| | | (0.772) | |
| Log Deaths Last 7 days × Non-essential shops closed | | | 6.965*** |
| | | | (0.857) |
| Observations | 754,739 | 754,364 | 603,499 |
| Adjusted $R^2$ | 0.56 | 0.85 | 0.84 |
| Mean Dependent Variable | 50.3 | 50.3 | 50.0 |
| **B: Responsiveness to New Cases** | | | |
| Log Cases Last 7 days | 3.480*** | 2.672*** | 2.517*** |
| | (0.333) | (0.311) | (0.354) |
| Log Cases Last 7 days × Lockdown | | 4.596*** | |
| | | (0.273) | |
| Log Cases Last 7 days × Non-essential shops closed | | | 4.965*** |
| | | | (0.342) |
| Observations | 753,979 | 753,979 | 603,155 |
| Adjusted $R^2$ | 0.85 | 0.85 | 0.84 |
| Mean Dependent Variable | 50.3 | 50.3 | 50.0 |
| **C: Responsiveness to Cumulative Deaths** | | | |
| Log Cumulative Deaths | 3.392*** | 2.825*** | 2.972*** |
| | (0.392) | (0.370) | (0.436) |
| Log Cumulative Deaths × Lockdown | | 4.100*** | |
| | | (0.252) | |
| Log Cumulative Deaths × Non-essential shops closed | | | 3.953*** |
| | | | (0.303) |
| Observations | 754,489 | 754,489 | 603,589 |
| Adjusted $R^2$ | 0.85 | 0.85 | 0.84 |
| Mean Dependent Variable | 50.3 | 50.3 | 50.0 |
| **D: Responsiveness to Cumulative Cases** | | | |
| Log Cumulative Cases | 5.105*** | 4.226*** | 4.063*** |
| | (0.399) | (0.389) | (0.428) |
| Log Cumulative Cases × Lockdown | | 2.845*** | |
| | | (0.164) | |
| Log Cumulative Cases × Non-essential shops closed | | | 3.267*** |
| | | | (0.222) |
| Observations | 754,489 | 754,489 | 603,589 |
| Adjusted $R^2$ | 0.85 | 0.85 | 0.85 |
| Mean Dependent Variable | 50.3 | 50.3 | 50.0 |
| County fixed effect | X | X | X |
| State × Day fixed effect | X | X | X |
| Outbreak time fixed effect | X | X | X |

*Notes:* Standard errors clustered at the county level are in parentheses. Significance levels: * 10%, ** 5%, *** 1%. Each regression includes county, state × day and outbreak time fixed effects. Social distancing (DEX) measures the percentage reduction in exposure to others at commercial venues compared to January-February 2020. See main text for more details. Unit of observation: county-day. Sample period: January 21, 2020 - January 29, 2021. Outbreak time is measured as the time since the first 3 reported COVID-19 deaths in the county. Deaths and cases due to COVID-19 are measured using a moving average over the last 7 days.

## TABLE S4: **Citizen Responsiveness To COVID-19: Time Outside Home**

| | (1) | (2) | (3) |
|---|---|---|---|
| | *Dependent variable: Social Distancing (MEI)* | | |
| **A: Responsiveness to New Deaths** | | | |
| Log Deaths Last 7 days | 11.51*** | 2.868*** | 3.648*** |
| | (0.282) | (0.143) | (0.186) |
| Log Deaths Last 7 days × Lockdown | | 1.826*** | |
| | | (0.436) | |
| Log Deaths Last 7 days × Non-essential shops closed | | | 0.944** |
| | | | (0.429) |
| Observations | 1,124,823 | 1,124,450 | 901,375 |
| Adjusted $R^2$ | 0.21 | 0.85 | 0.86 |
| Mean Dependent Variable | 22.4 | 22.4 | 22.5 |
| **B: Responsiveness to New Cases** | | | |
| Log Cases Last 7 days | 2.420*** | 2.259*** | 2.111*** |
| | (0.0734) | (0.0695) | (0.0781) |
| Log Cases Last 7 days × Lockdown | | 1.182*** | |
| | | (0.155) | |
| Log Cases Last 7 days × Non-essential shops closed | | | 1.140*** |
| | | | (0.147) |
| Observations | 1,123,698 | 1,123,698 | 900,849 |
| Adjusted $R^2$ | 0.85 | 0.85 | 0.87 |
| Mean Dependent Variable | 22.4 | 22.4 | 22.5 |
| **C: Responsiveness to Cumulative Deaths** | | | |
| Log Cumulative Deaths | 1.734*** | 1.567*** | 1.665*** |
| | (0.0864) | (0.0851) | (0.0983) |
| Log Cumulative Deaths × Lockdown | | 1.412*** | |
| | | (0.136) | |
| Log Cumulative Deaths × Non-essential shops closed | | | 1.062*** |
| | | | (0.121) |
| Observations | 1,124,582 | 1,124,582 | 901,472 |
| Adjusted $R^2$ | 0.85 | 0.85 | 0.86 |
| Mean Dependent Variable | 22.4 | 22.4 | 22.5 |
| **D: Responsiveness to Cumulative Cases** | | | |
| Log Cumulative Cases | 2.208*** | 2.003*** | 2.004*** |
| | (0.0764) | (0.0742) | (0.0807) |
| Log Cumulative Cases × Lockdown | | 0.810*** | |
| | | (0.0869) | |
| Log Cumulative Cases × Non-essential shops closed | | | 0.761*** |
| | | | (0.0801) |
| Observations | 1,124,582 | 1,124,582 | 901,472 |
| Adjusted $R^2$ | 0.85 | 0.85 | 0.87 |
| Mean Dependent Variable | 22.4 | 22.4 | 22.5 |
| County fixed effect | X | X | X |
| State × Day fixed effect | X | X | X |
| Outbreak time fixed effect | X | X | X |

*Notes:* Standard errors clustered at the county level are in parentheses. Significance levels: * 10%, ** 5%, *** 1%. Each regression includes county, state × day and outbreak time fixed effects. Social distancing (MEI) measures the percentage reduction in time outside home compared to January-February 2020. See main text for more details. Unit of observation: county-day. Sample period: January 21, 2020 - January 29, 2021. Outbreak time is measured as the time since the first 3 reported COVID-19 deaths in the county. Deaths and cases due to COVID-19 are measured using a moving average over the last 7 days.

# TABLE S5: **Robustness on Responsiveness To COVID-19**

| | (1) Index | (2) Index | (3) Index | (4) DEX | (5) DEX | (6) DEX | (7) MEI | (8) MEI | (9) MEI |
|---|---|---|---|---|---|---|---|---|---|
| | *Dependent variable: Social Distancing* | | | | | | | | |
| **A: Responsiveness to Deaths in Last 14 Days** | | | | | | | | | |
| Log Deaths Last 14 days | 5.446*** | 4.248*** | 5.499*** | 7.397*** | 5.397*** | 6.978*** | 3.800*** | 3.388*** | 4.278*** |
| | (0.392) | (0.360) | (0.533) | (0.706) | (0.667) | (1.011) | (0.203) | (0.170) | (0.216) |
| Lockdown × Log Deaths Last 14 days | | 4.923*** | | | 8.265*** | | | 1.780*** | |
| | | (0.545) | | | (0.816) | | | (0.450) | |
| Log Deaths Last 14 days × Non-essential Shops Closed | | | 3.768*** | | | 6.944*** | | | 0.744* |
| | | | (0.582) | | | (0.946) | | | (0.447) |
| Observations | 750,369 | 750,369 | 601,505 | 754,393 | 754,393 | 603,517 | 1,124,486 | 1,124,486 | 901,400 |
| Adjusted $R^2$ | 0.88 | 0.88 | 0.87 | 0.85 | 0.85 | 0.84 | 0.85 | 0.85 | 0.86 |
| Mean Dependent Variable | 37.6 | 37.6 | 37.4 | 50.3 | 50.3 | 50.0 | 22.4 | 22.4 | 22.5 |
| **B: Responsiveness to Cases in Last 14 Days** | | | | | | | | | |
| Log Cases Last 14 days | 3.206*** | 2.702*** | 2.569*** | 3.796*** | 2.958*** | 2.802*** | 2.625*** | 2.454*** | 2.311*** |
| | (0.197) | (0.182) | (0.206) | (0.360) | (0.336) | (0.381) | (0.0799) | (0.0758) | (0.0853) |
| Lockdown × Log Cases Last 14 days | | 2.957*** | | | 4.915*** | | | 1.290*** | |
| | | (0.193) | | | (0.288) | | | (0.162) | |
| Log Cases Last 14 days × Non-essential Shops Closed | | | 3.111*** | | | 5.285*** | | | 1.214*** |
| | | | (0.224) | | | (0.362) | | | (0.154) |
| Observations | 750,125 | 750,125 | 601,268 | 754,145 | 754,145 | 603,280 | 1,124,026 | 1,124,026 | 901,102 |
| Adjusted $R^2$ | 0.88 | 0.88 | 0.88 | 0.85 | 0.85 | 0.84 | 0.85 | 0.85 | 0.87 |
| Mean Dependent Variable | 37.6 | 37.6 | 37.4 | 50.3 | 50.3 | 50.0 | 22.4 | 22.4 | 22.5 |
| **C: Responsiveness to Deaths in Last 21 Days** | | | | | | | | | |
| Log Deaths Last 21 days | 5.776*** | 4.556*** | 5.836*** | 7.856*** | 5.828*** | 7.433*** | 4.009*** | 3.587*** | 4.502*** |
| | (0.416) | (0.382) | (0.560) | (0.749) | (0.711) | (1.065) | (0.216) | (0.183) | (0.231) |
| Log Deaths Last 21 days × Lockdown | | 5.026*** | | | 8.408*** | | | 1.829*** | |
| | | (0.564) | | | (0.847) | | | (0.459) | |
| Log Deaths Last 21 days × Non-essential Shops Closed | | | 3.822*** | | | 7.060*** | | | 0.716 |
| | | | (0.615) | | | (1.006) | | | (0.457) |
| Observations | 750,380 | 750,380 | 601,493 | 754,404 | 754,404 | 603,505 | 1,124,497 | 1,124,497 | 901,388 |
| Adjusted $R^2$ | 0.88 | 0.88 | 0.88 | 0.85 | 0.85 | 0.84 | 0.85 | 0.85 | 0.86 |
| Mean Dependent Variable | 37.6 | 37.6 | 37.4 | 50.3 | 50.3 | 50.0 | 22.4 | 22.4 | 22.5 |
| **D: Responsiveness to Cases in Last 21 Days** | | | | | | | | | |
| Log Cases Last 21 days | 3.317*** | 2.807*** | 2.695*** | 3.912*** | 3.073*** | 2.944*** | 2.692*** | 2.516*** | 2.393*** |
| | (0.204) | (0.189) | (0.213) | (0.374) | (0.349) | (0.396) | (0.0848) | (0.0804) | (0.0908) |
| Log Cases Last 21 days × Lockdown | | 3.157*** | | | 5.199*** | | | 1.403*** | |
| | | (0.201) | | | (0.301) | | | (0.169) | |
| Log Cases Last 21 days × Non-essential Shops Closed | | | 3.267*** | | | 5.519*** | | | 1.286*** |
| | | | (0.235) | | | (0.377) | | | (0.161) |
| Observations | 750,229 | 750,229 | 601,347 | 754,253 | 754,253 | 603,359 | 1,124,255 | 1,124,255 | 901,217 |
| Adjusted $R^2$ | 0.88 | 0.88 | 0.88 | 0.85 | 0.85 | 0.84 | 0.85 | 0.85 | 0.87 |
| Mean Dependent Variable | 37.6 | 37.6 | 37.4 | 50.3 | 50.3 | 50.0 | 22.4 | 22.4 | 22.5 |
| County fixed effect | X | X | X | X | X | X | X | X | X |
| State × Day fixed effect | X | X | X | X | X | X | X | X | X |
| Outbreak time fixed effect | X | X | X | X | X | X | X | X | X |

*Notes:* Standard errors clustered at the county level are in parentheses. Significance levels: * 10%, ** 5%, *** 1%. Each regression includes county, state × day and outbreak time fixed effects. The social distancing index measures the average percentage reduction in time outside home and exposure to others at commercial venues compared to January-February 2020. See main text for more details. Unit of observation: county-day. Sample period: January 21, 2020 - January 29, 2021. Outbreak time is measured as the time since the first 3 reported COVID-19 deaths in the county. Deaths and cases due to COVID-19 are measured using a moving average over the last 14 and 21 days (Panels A-B and C-D respectively).

TABLE S6: **Determinants of Responsiveness To COVID-19 Outbreaks: Exposure to Others**

| | (1) | (2) | (3) | (4) | (5) | (6) | (7) | (8) |
|---|---|---|---|---|---|---|---|---|
| | *Dependent variable: Social Distancing (DEX)* | | | | | | | |
| **A: Responsiveness to New Deaths** | | | | | | | | |
| Log Deaths Last 7 days | 2.077*** | 2.475*** | 2.826*** | 1.784*** | 1.082** | 1.873*** | 2.831*** | 0.0969 |
| | (0.416) | (0.430) | (0.490) | (0.416) | (0.442) | (0.475) | (0.444) | (0.512) |
| Log Deaths Last 7 days × % Aged 65 Years and Older | -1.457*** | | | | | | | -1.472** |
| | (0.471) | | | | | | | (0.685) |
| Log Deaths Last 7 days × % with 3+ Risk Factors for COVID-19 | | -0.838 | | | | | | 0.886 |
| | | (0.682) | | | | | | (1.012) |
| Log Deaths Last 7 days × % without Health Insurance | | | 1.007 | | | | | 2.445*** |
| | | | (0.721) | | | | | (0.828) |
| Log Deaths Last 7 days × Median Household Income | | | | 1.812*** | | | | 1.529** |
| | | | | (0.400) | | | | (0.721) |
| Log Deaths Last 7 days × % with Bachelor's Degree | | | | | 2.677*** | | | 2.178*** |
| | | | | | (0.482) | | | (0.717) |
| Log Deaths Last 7 days × % Vote Democrat 2016 | | | | | | 1.797*** | | 1.013 |
| | | | | | | (0.575) | | (0.793) |
| Log Deaths Last 7 days × Social Capital Index | | | | | | | 0.949 | -1.609 |
| | | | | | | | (1.066) | (1.186) |
| Observations | 754,364 | 754,364 | 754,364 | 754,364 | 754,364 | 752,489 | 750,614 | 748,739 |
| Adjusted $R^2$ | 0.85 | 0.85 | 0.85 | 0.85 | 0.85 | 0.85 | 0.85 | 0.85 |
| Mean Dependent Variable | 50.3 | 50.3 | 50.3 | 50.3 | 50.3 | 50.3 | 50.3 | 50.2 |
| **B: Responsiveness to New Cases** | | | | | | | | |
| Log Cases Last 7 days | 2.002*** | 2.096*** | 2.169*** | 1.960*** | 1.690*** | 1.934*** | 2.282*** | 1.499*** |
| | (0.252) | (0.266) | (0.265) | (0.242) | (0.242) | (0.250) | (0.249) | (0.222) |
| Log Cases Last 7 days × % Aged 65 Years and Older | -0.286** | | | | | | | -0.307 |
| | (0.127) | | | | | | | (0.205) |
| Log Cases Last 7 days × % with 3+ Risk Factors for COVID-19 | | -0.229 | | | | | | 0.316 |
| | | (0.162) | | | | | | (0.344) |
| Log Cases Last 7 days × % without Health Insurance | | | 0.0908 | | | | | 0.666** |
| | | | (0.286) | | | | | (0.335) |
| Log Cases Last 7 days × Median Household Income | | | | 0.563*** | | | | 0.423 |
| | | | | (0.144) | | | | (0.268) |
| Log Cases Last 7 days × % with Bachelor's Degree | | | | | 0.878*** | | | 0.642*** |
| | | | | | (0.149) | | | (0.228) |
| Log Cases Last 7 days × % Vote Democrat 2016 | | | | | | 0.975*** | | 0.556** |
| | | | | | | (0.191) | | (0.248) |
| Log Cases Last 7 days × Social Capital Index | | | | | | | 0.435* | -0.175 |
| | | | | | | | (0.259) | (0.297) |
| Observations | 753,979 | 753,979 | 753,979 | 753,979 | 753,979 | 752,104 | 750,230 | 748,355 |
| Adjusted $R^2$ | 0.85 | 0.85 | 0.85 | 0.85 | 0.86 | 0.85 | 0.85 | 0.86 |
| Mean Dependent Variable | 50.3 | 50.3 | 50.3 | 50.3 | 50.3 | 50.3 | 50.3 | 50.2 |
| County fixed effect | X | X | X | X | X | X | X | X |
| State × Day fixed effect | X | X | X | X | X | X | X | X |
| Outbreak time fixed effect | X | X | X | X | X | X | X | X |
| Interaction with Demographic Characteristics | X | X | X | X | X | X | X | X |

*Notes:* Standard errors clustered at the state level are in parentheses. Significance levels: * 10%, ** 5%, *** 1%. Each regression includes county, state × day and outbreak time fixed effects. Social distancing (DEX) measures the percentage reduction in exposure to others at commercial venues compared to January-February 2020. See main text for more details. Unit of observation: county-day. Sample period: January 21, 2020 - January 29, 2021. Outbreak time is measured as the time since the first 3 reported COVID-19 deaths in the county. Deaths and cases due to COVID-19 are measured using a moving average over the last 7 days.

## TABLE S7: **Determinants of Responsiveness To COVID-19 outbreaks: Time Outside Home**

| | (1) | (2) | (3) | (4) | (5) | (6) | (7) | (8) |
|---|---|---|---|---|---|---|---|---|
| | | | | *Dependent variable: Social Distancing (MEI)* | | | | |
| **A: Responsiveness to New Deaths** | | | | | | | | |
| Log Deaths Last 7 days | 2.702*** | 2.759*** | 2.708*** | 2.195*** | 1.972*** | 2.243*** | 3.286*** | 1.779*** |
| | (0.163) | (0.157) | (0.145) | (0.139) | (0.139) | (0.151) | (0.153) | (0.146) |
| Log Deaths Last 7 days × % Aged 65 Years and Older | -0.504*** | | | | | | | 0.275* |
| | (0.159) | | | | | | | (0.162) |
| Log Deaths Last 7 days × % with 3+ Risk Factors for COVID-19 | | -1.149*** | | | | | | -0.416* |
| | | (0.181) | | | | | | (0.227) |
| Log Deaths Last 7 days × % without Health Insurance | | | -0.688*** | | | | | 0.444*** |
| | | | (0.195) | | | | | (0.170) |
| Log Deaths Last 7 days × Median Household Income | | | | 1.765*** | | | | 1.377*** |
| | | | | (0.104) | | | | (0.204) |
| Log Deaths Last 7 days × % with Bachelor's Degree | | | | | 1.785*** | | | 0.505** |
| | | | | | (0.117) | | | (0.205) |
| Log Deaths Last 7 days × % Vote Democrat 2016 | | | | | | 1.698*** | | 1.208*** |
| | | | | | | (0.190) | | (0.214) |
| Log Deaths Last 7 days × Social Capital Index | | | | | | | 1.559*** | -0.259 |
| | | | | | | | (0.185) | (0.207) |
| Observations | 1,124,450 | 1,124,450 | 1,124,450 | 1,124,450 | 1,124,450 | 1,118,482 | 1,091,999 | 1,086,404 |
| Adjusted $R^2$ | 0.85 | 0.85 | 0.85 | 0.85 | 0.85 | 0.85 | 0.86 | 0.86 |
| Mean Dependent Variable | 22.4 | 22.4 | 22.4 | 22.4 | 22.4 | 22.4 | 22.5 | 22.5 |
| **B: Responsiveness to New Cases** | | | | | | | | |
| Log Cases Last 7 days | 2.119*** | 2.143*** | 2.170*** | 2.074*** | 1.962*** | 2.042*** | 2.255*** | 1.942*** |
| | (0.0699) | (0.0695) | (0.0680) | (0.0676) | (0.0662) | (0.0660) | (0.0693) | (0.0657) |
| Log Cases Last 7 days × % Aged 65 Years and Older | -0.138*** | | | | | | | 0.0568 |
| | (0.0436) | | | | | | | (0.0497) |
| Log Cases Last 7 days × % with 3+ Risk Factors for COVID-19 | | -0.209*** | | | | | | 0.0244 |
| | | (0.0446) | | | | | | (0.0592) |
| Log Cases Last 7 days × % without Health Insurance | | | -0.589*** | | | | | -0.244*** |
| | | | (0.0634) | | | | | (0.0609) |
| Log Cases Last 7 days × Median Household Income | | | | 0.457*** | | | | 0.331*** |
| | | | | (0.0376) | | | | (0.0558) |
| Log Cases Last 7 days × % with Bachelor's Degree | | | | | 0.560*** | | | 0.153*** |
| | | | | | (0.0350) | | | (0.0501) |
| Log Cases Last 7 days × % Vote Democrat 2016 | | | | | | 0.937*** | | 0.671*** |
| | | | | | | (0.0556) | | (0.0656) |
| Log Cases Last 7 days × Social Capital Index | | | | | | | 0.314*** | -0.0881 |
| | | | | | | | (0.0576) | (0.0571) |
| Observations | 1,123,698 | 1,123,698 | 1,123,698 | 1,123,698 | 1,123,698 | 1,117,730 | 1,091,305 | 1,085,710 |
| Adjusted $R^2$ | 0.85 | 0.85 | 0.86 | 0.86 | 0.86 | 0.86 | 0.86 | 0.86 |
| Mean Dependent Variable | 22.4 | 22.4 | 22.4 | 22.4 | 22.4 | 22.4 | 22.5 | 22.5 |
| County fixed effect | X | X | X | X | X | X | X | X |
| State × Day fixed effect | X | X | X | X | X | X | X | X |
| Outbreak time fixed effect | X | X | X | X | X | X | X | X |
| Interaction with Demographic Characteristics | X | X | X | X | X | X | X | X |

*Notes:* Standard errors clustered at the state level are in parentheses. Significance levels: * 10%, ** 5%, *** 1%. Each regression and includes county, state × day and outbreak time fixed effects. Social distancing (MEI) measures the percentage reduction in time outside home compared to January-February 2020. See main text for more details. Unit of observation: county-day. Sample period: January 21, 2020 - January 29, 2021. Outbreak time is measured as the time since the first 3 reported COVID-19 deaths in the county. Deaths and cases due to COVID-19 are measured using a moving average over the last 7 days.

TABLE S8: **Determinants of Social Distancing: Alternative Social Capital Measures**

| | (1) | (2) | (3) | (4) | (5) | (6) |
|---|---|---|---|---|---|---|
| | *Dependent variable: Social Distancing Index* | | | | | |
| Social Capital Index | 1.821*** | 1.911*** | | | | |
| | (0.467) | (0.570) | | | | |
| Social Capital (Rupashingha et al.) | | | 0.781 | 0.211 | | |
| | | | (0.488) | (0.306) | | |
| Voter Participation | | | | | 1.989*** | 1.034** |
| | | | | | (0.356) | (0.524) |
| % Aged 65 Years and Older | | 1.654*** | | 1.833*** | | 1.497*** |
| | | (0.427) | | (0.425) | | (0.442) |
| % with 3+ Risk Factors for COVID-19 | | 1.479*** | | 1.315** | | 1.363*** |
| | | (0.538) | | (0.520) | | (0.521) |
| % without Health Insurance | | 0.181 | | 0.180 | | 0.237 |
| | | (0.674) | | (0.670) | | (0.663) |
| Median Household Income | | 1.517*** | | 2.097*** | | 1.645*** |
| | | (0.495) | | (0.521) | | (0.515) |
| % with Bachelor's Degree | | -1.375** | | -1.193** | | -1.337** |
| | | (0.553) | | (0.537) | | (0.557) |
| % Vote Democrat 2016 | | 2.858*** | | 2.753*** | | 2.626*** |
| | | (0.543) | | (0.543) | | (0.537) |
| Log Deaths Last 7 days | 2.843*** | 1.760*** | 2.704*** | 1.899*** | 2.769*** | 1.814*** |
| | (0.506) | (0.456) | (0.508) | (0.469) | (0.507) | (0.463) |
| Log Cases Last 7 days | 1.709*** | 2.100*** | 1.706*** | 2.077*** | 1.736*** | 2.064*** |
| | (0.185) | (0.179) | (0.187) | (0.180) | (0.185) | (0.181) |
| Observations | 746,103 | 744,238 | 749,832 | 747,967 | 747,967 | 747,967 |
| Adjusted $R^2$ | 0.74 | 0.75 | 0.74 | 0.75 | 0.75 | 0.75 |
| Mean Dependent Variable | 37.6 | 37.5 | 37.6 | 37.6 | 37.6 | 37.6 |
| State $\times$ Day fixed effect | X | X | X | X | X | X |
| Outbreak time fixed effect | X | X | X | X | X | X |
| Demographic Characteristics | X | X | X | X | X | X |

*Notes:* Standard errors clustered at the county level are in parentheses. Significance levels: * 10%, ** 5%, *** 1%. Our preferred measure of social capital is developed by the Social Capital Project from the U.S. Joint Economic Committee, and their county index uses a principal component analysis of four categories of variables : (1) family unity (2) community health (3) institutional health (4) collective efficacy. The social capital index 2014 comes from the updated 2014 measure developed by Rupasingha, Goetz and Freshwater (2006) which uses a principal component analysis to include four social capital factors: (1) The aggregate of various civic, religious, business, labor, political associations in the county divided by population per 1000; (2) Voter turnout in the 2012 election; (3) Census response rate; (4) Number of non-profit organizations excluding those with an international approach. Voter participation is a measure of civic capital as used in Barrios et al. (2020) and is the average voter participation in counties at presidential elections between 2004 and 2016. The social distancing index measures the average percentage reduction in time outside home and exposure to others at commercial venues compared to January-February 2020. See main text for more details. Unit of observation: county-day. Sample period: January 21, 2020 - January 29, 2021. Outbreak time is measured as the time since the first 3 reported COVID-19 deaths in the county. Deaths and cases due to COVID-19 are measured using a moving average over the last 7 days.

## TABLE S9: **Determinants of Responsiveness To COVID-19 outbreaks: Alternative Social Capital Measures**

| | (1) | (2) | (3) | (4) | (5) | (6) |
|---|---|---|---|---|---|---|
| | | | *Dependent variable: Social Distancing Index* | | | |
| **A: Responsiveness to New Deaths** | | | | | | |
| Log Deaths Last 7 days | 2.909*** | 0.742*** | 2.173*** | 0.497** | 2.516*** | 0.925*** |
| | (0.259) | (0.278) | (0.263) | (0.237) | (0.234) | (0.247) |
| Log Deaths Last 7 days × Social Capital Index | 1.364** | -0.924 | | | | |
| | (0.551) | (0.609) | | | | |
| Log Deaths Last 7 days × Social Capital (Rupashingha et al.) | | | -0.975** | -1.560*** | | |
| | | | (0.491) | (0.463) | | |
| Log Deaths Last 7 days × Voter Participation | | | | | 1.322*** | -0.751** |
| | | | | | (0.255) | (0.365) |
| Log Deaths Last 7 days × % Aged 65 Years and Older | | -0.470 | | -0.392 | | -0.415 |
| | | (0.362) | | (0.379) | | (0.407) |
| Log Deaths Last 7 days × % with 3+ Risk Factors for COVID-19 | | 0.243 | | 0.372 | | 0.428 |
| | | (0.539) | | (0.564) | | (0.586) |
| Log Deaths Last 7 days × % without Health Insurance | | 1.480*** | | 1.306*** | | 1.318*** |
| | | (0.437) | | (0.440) | | (0.441) |
| Log Deaths Last 7 days × Median Household Income | | 1.503*** | | 1.046*** | | 1.397*** |
| | | (0.393) | | (0.348) | | (0.341) |
| Log Deaths Last 7 days × % with Bachelor's Degree | | 1.322*** | | 1.507*** | | 1.445*** |
| | | (0.377) | | (0.362) | | (0.404) |
| Log Deaths Last 7 days × % Vote Democrat 2016 | | 1.048** | | 1.032** | | 1.198*** |
| | | (0.427) | | (0.428) | | (0.436) |
| Observations | 746,610 | 744,745 | 750,340 | 748,475 | 748,475 | 748,475 |
| Adjusted $R^2$ | 0.88 | 0.88 | 0.88 | 0.88 | 0.88 | 0.88 |
| Mean Dependent Variable | 37.6 | 37.5 | 37.6 | 37.6 | 37.6 | 37.6 |
| **B: Responsiveness to New Cases** | | | | | | |
| Log Cases Last 7 days | 2.248*** | 1.621*** | 2.062*** | 1.558*** | 2.178*** | 1.531*** |
| | (0.139) | (0.122) | (0.134) | (0.124) | (0.139) | (0.131) |
| Log Cases Last 7 days × Social Capital Index | 0.458*** | -0.130 | | | | |
| | (0.134) | (0.153) | | | | |
| Log Cases Last 7 days × Social Capital (Rupashingha et al.) | | | -0.137* | -0.299** | | |
| | | | (0.0812) | (0.117) | | |
| Log Cases Last 7 days × Voter Participation | | | | | 0.358*** | -0.478*** |
| | | | | | (0.0808) | (0.112) |
| Log Cases Last 7 days × % Aged 65 Years and Older | | -0.0948 | | -0.0502 | | 0.0469 |
| | | (0.106) | | (0.109) | | (0.111) |
| Log Cases Last 7 days × % with 3+ Risk Factors for COVID-19 | | 0.168 | | 0.178 | | 0.161 |
| | | (0.174) | | (0.177) | | (0.178) |
| Log Cases Last 7 days × % without Health Insurance | | 0.245 | | 0.228 | | 0.176 |
| | | (0.174) | | (0.173) | | (0.175) |
| Log Cases Last 7 days × Median Household Income | | 0.407*** | | 0.348*** | | 0.547*** |
| | | (0.142) | | (0.130) | | (0.134) |
| Log Cases Last 7 days × % with Bachelor's Degree | | 0.404*** | | 0.446*** | | 0.479*** |
| | | (0.117) | | (0.116) | | (0.122) |
| Log Cases Last 7 days × % Vote Democrat 2016 | | 0.591*** | | 0.578*** | | 0.692*** |
| | | (0.131) | | (0.129) | | (0.127) |
| Observations | 746,228 | 744,363 | 749,957 | 748,092 | 748,092 | 748,092 |
| Adjusted $R^2$ | 0.88 | 0.88 | 0.88 | 0.88 | 0.88 | 0.88 |
| Mean Dependent Variable | 37.6 | 37.5 | 37.6 | 37.6 | 37.6 | 37.6 |
| County fixed effect | X | X | X | X | X | X |
| State × Day fixed effect | X | X | X | X | X | X |
| Outbreak time fixed effect | X | X | X | X | X | X |
| Interaction with Demographic Characteristics | X | X | X | X | X | X |

*Notes:* Standard errors clustered at the state level are in parentheses. Significance levels: * 10%, ** 5%, *** 1%. Each regression includes county, state × day and outbreak time fixed effects. Our preferred measure of social capital is developed by the Social Capital Project from the U.S. Joint Economic Committee, and their county index uses a principal component analysis of four categories of variables : (1) family unity (2) community health (3) institutional health (4) collective efficacy. The social capital index 2014 comes from the updated 2014 measure developed by Rupasingha, Goetz and Freshwater (2006) which uses a principal component analysis to include four social capital factors: (1) The aggregate of various civic, religious, business, labor, political associations in the county divided by population per 1000; (2) Voter turnout in the 2012 election; (3) Census response rate; (4) Number of non-profit organizations excluding those with an international approach. Voter participation is a measure of civic capital as used in Barrios et al. (2020) and is the average voter participation in counties at presidential elections between 2004 and 2016. The social distancing index measures the average percentage reduction in time outside home and exposure to others at commercial venues compared to January-February 2020. See main text for more details. Unit of observation: county-day. Sample period: January 21, 2020 - January 29, 2021. Outbreak time is measured as the time since the first 3 reported COVID-19 deaths in the county. Deaths and cases due to COVID-19 are measured using a moving average over the last 7 days.

TABLE S10: **Determinants of State Government Responsiveness To COVID-19**

| | (1) | (2) | (3) | (4) |
|---|---|---|---|---|
| | Lockdown | Non-Essential Shops Closed | Lockdown | Non-Essential Shops Closed |
| Log Deaths Last 7 Days | 0.0560*** | 0.0357* | | |
| | (0.0103) | (0.0199) | | |
| Log Deaths Last 7 Days × Share vote Democrat 2016 | -0.00784 | -0.00303 | | |
| | (0.00678) | (0.0188) | | |
| Log Deaths Last 7 Days × Lame Duck Governor | 0.0000350 | -0.0249 | | |
| | (0.00787) | (0.0203) | | |
| Log Deaths Last 7 Days × Pro-Trump Governor | -0.0139* | -0.0203 | | |
| | (0.00712) | (0.0136) | | |
| Log Deaths Last 7 Days × Governor with Science Background | -0.00826 | 0.0351** | | |
| | (0.00566) | (0.0175) | | |
| Log Deaths Last 7 Days × Mortality rate from Deaths of despair | -0.0133*** | -0.00282 | | |
| | (0.00481) | (0.0112) | | |
| Log Deaths Last 7 Days × % in Fair or Poor Health | -0.0110 | -0.00264 | | |
| | (0.00661) | (0.0191) | | |
| Log Deaths Last 7 Days × Centralized Public Health Governance | 0.0201** | 0.0131 | | |
| | (0.00777) | (0.0209) | | |
| Log Deaths Last 7 Days × Social Capital index | -0.0160** | 0.0142 | | |
| | (0.00603) | (0.0209) | | |
| Log Deaths Last 7 Days × % with Bachelor's Degree | -0.00178 | -0.0272 | | |
| | (0.00755) | (0.0281) | | |
| Log Deaths Last 7 Days × State and Local Government Spending Per Capita | -0.00236 | 0.0162 | | |
| | (0.00456) | (0.0175) | | |
| Log Deaths Last 7 Days × % Essential Workers | 0.00135 | 0.00940 | | |
| | (0.00740) | (0.0147) | | |
| Log Cases Last 7 Days | | | 0.0274** | 0.00714 |
| | | | (0.0117) | (0.0195) |
| Log Cases Last 7 Days × Share vote Democrat 2016 | | | -0.000201 | -0.00774 |
| | | | (0.00264) | (0.00824) |
| Log Cases Last 7 Days × Lame Duck Governor | | | -0.00114 | -0.00864 |
| | | | (0.00201) | (0.00780) |
| Log Cases Last 7 Days × Pro-Trump Governor | | | -0.00246 | -0.00617 |
| | | | (0.00217) | (0.00468) |
| Log Cases Last 7 Days × Governor with Science Background | | | -0.00225 | 0.00773 |
| | | | (0.00183) | (0.00815) |
| Log Cases Last 7 Days × Mortality rate from Deaths of despair | | | -0.00509*** | -0.00705* |
| | | | (0.00180) | (0.00409) |
| Log Cases Last 7 Days × % in Fair or Poor Health | | | 0.000602 | 0.000606 |
| | | | (0.00205) | (0.00680) |
| Log Cases Last 7 Days × Centralized Public Health Governance | | | 0.00569* | 0.00196 |
| | | | (0.00284) | (0.00712) |
| Log Cases Last 7 Days × Social Capital index | | | -0.00402* | -0.00388 |
| | | | (0.00209) | (0.00813) |
| Log Cases Last 7 Days × % with Bachelor's Degree | | | 0.00362 | -0.00436 |
| | | | (0.00222) | (0.00969) |
| Log Cases Last 7 Days × State and Local Government Spending Per Capita | | | -0.00312 | 0.0247** |
| | | | (0.00267) | (0.0105) |
| Log Cases Last 7 Days × % Essential Workers | | | 0.000634 | -0.00429 |
| | | | (0.00302) | (0.00655) |
| Observations | 19,147 | 14,950 | 19,155 | 14,955 |
| Adjusted $R^2$ | 0.71 | 0.74 | 0.71 | 0.74 |
| State fixed effect | X | X | X | X |
| Day fixed effect | X | X | X | X |
| Outbreak time fixed effect | X | X | X | X |
| Interaction with Demographic Characteristics | X | X | X | X |

*Notes:* Standard errors clustered at the county level are in parentheses. Significance levels: * 10%, ** 5%, *** 1%. Each regression includes state, day and outbreak time fixed effects. Unit of observation: state-day. Outbreak time is measured as the time since the first 3 reported COVID-19 deaths in the county. Deaths and cases due to COVID-19 are measured using a moving average over the last 7 days. Lockdown is a dummy variable indicating that a curfew or lockdown are in place for at least part of the day. See main text for details.

TABLE S11: **Robustness on State Government Responsiveness To COVID-19**

| | (1) | (2) | (3) | (4) |
|---|---|---|---|---|
| | Lockdown | Non-Essential Shops Closed | Limit Gathering in Public Space | NPI Index |
| **A: Responsiveness to Deaths in Last 14 Days** | | | | |
| Log Deaths Last 14 Days | 0.0540*** | 0.0343 | 0.0692* | 0.145*** |
| | (0.0105) | (0.0234) | (0.0358) | (0.0420) |
| Observations | 19,630 | 15,262 | 19,356 | 15,262 |
| Adjusted $R^2$ | 0.71 | 0.73 | 0.53 | 0.87 |
| **B: Responsiveness to Cases in Last 14 Days** | | | | |
| Log Cases Last 14 Days | 0.0316*** | 0.0106 | 0.0517 | 0.0801** |
| | (0.0110) | (0.0189) | (0.0334) | (0.0373) |
| Observations | 19,616 | 15,248 | 19,342 | 15,248 |
| Adjusted $R^2$ | 0.71 | 0.73 | 0.53 | 0.87 |
| **C: Responsiveness to Deaths in Last 21 Days** | | | | |
| Log Deaths Last 21 Days | 0.0552*** | 0.0380 | 0.0692* | 0.149*** |
| | (0.0105) | (0.0241) | (0.0362) | (0.0435) |
| Observations | 19,630 | 15,262 | 19,356 | 15,262 |
| Adjusted $R^2$ | 0.71 | 0.73 | 0.53 | 0.87 |
| Mean Dependent Variable | 0.11 | 0.21 | 0.53 | 1.91 |
| **D: Responsiveness to Cases in Last 21 Days** | | | | |
| Log Cases Last 21 Days | 0.0345*** | 0.0128 | 0.0570 | 0.0897** |
| | (0.0109) | (0.0191) | (0.0342) | (0.0373) |
| Observations | 19,609 | 15,241 | 19,335 | 15,241 |
| Adjusted $R^2$ | 0.71 | 0.73 | 0.53 | 0.87 |
| State fixed effect | X | X | X | X |
| Day fixed effect | X | X | X | X |
| Outbreak time fixed effect | X | X | X | X |

*Notes:* Standard errors clustered at the state level are in parentheses. Significance levels: * 10%, ** 5%, *** 1%. Each regression includes state, day and outbreak time fixed effects. Unit of observation: state-day. Outbreak time is measured as the time since the first 3 reported COVID-19 deaths in the county. Deaths and cases due to COVID-19 are measured using a moving average over the last 14 and 21 days (Panels A-B and C-D respectively). Lockdown is a dummy variable indicating that a curfew or lockdown are in place for at least part of the day. The NPI index measures the intensity of movement restriction policy on a scale of 0 to 6. See main text for details.

# References

**Atkinson, Tyler, Jim Dolmas, Christoffer Koch, Evan F Koenig, Karel Mertens, Anthony Murphy, and Kei-Mu Yi.** 2020. *Mobility and Engagement Following the SARS-Cov-2 Outbreak.* Federal Reserve Bank of Dallas, Research Department.

**Barrios, John M, Efraim Benmelech, Yael V Hochberg, Paola Sapienza, and Luigi Zingales.** 2020. "Civic capital and social distancing during the Covid-19 pandemic." *Journal of Public Economics*, 193: 104310.

**Couture, Victor, Jonathan I Dingel, Allison E Green, Jessie Handbury, and Kevin R Williams.** 2020. "Measuring Movement and Social Contact with Smartphone Data: A Real-Time Applicationto COVID-19." National Bureau of Economic Research.

**Rupasingha, Anil, Stephan J Goetz, and David Freshwater.** 2006. "The production of social capital in US counties." *The Journal of Socio-Economics*, 35(1): 83–101.
