# Supplementary figures and images for: Pandemic responsiveness: Evidence from social distancing and lockdown policy during COVID-19
Source: PLoS One. 2022 May 19;17(5):e0267611. doi: 10.1371/journal.pone.0267611 (PMC9119550; doi:10.1371/journal.pone.0267611)

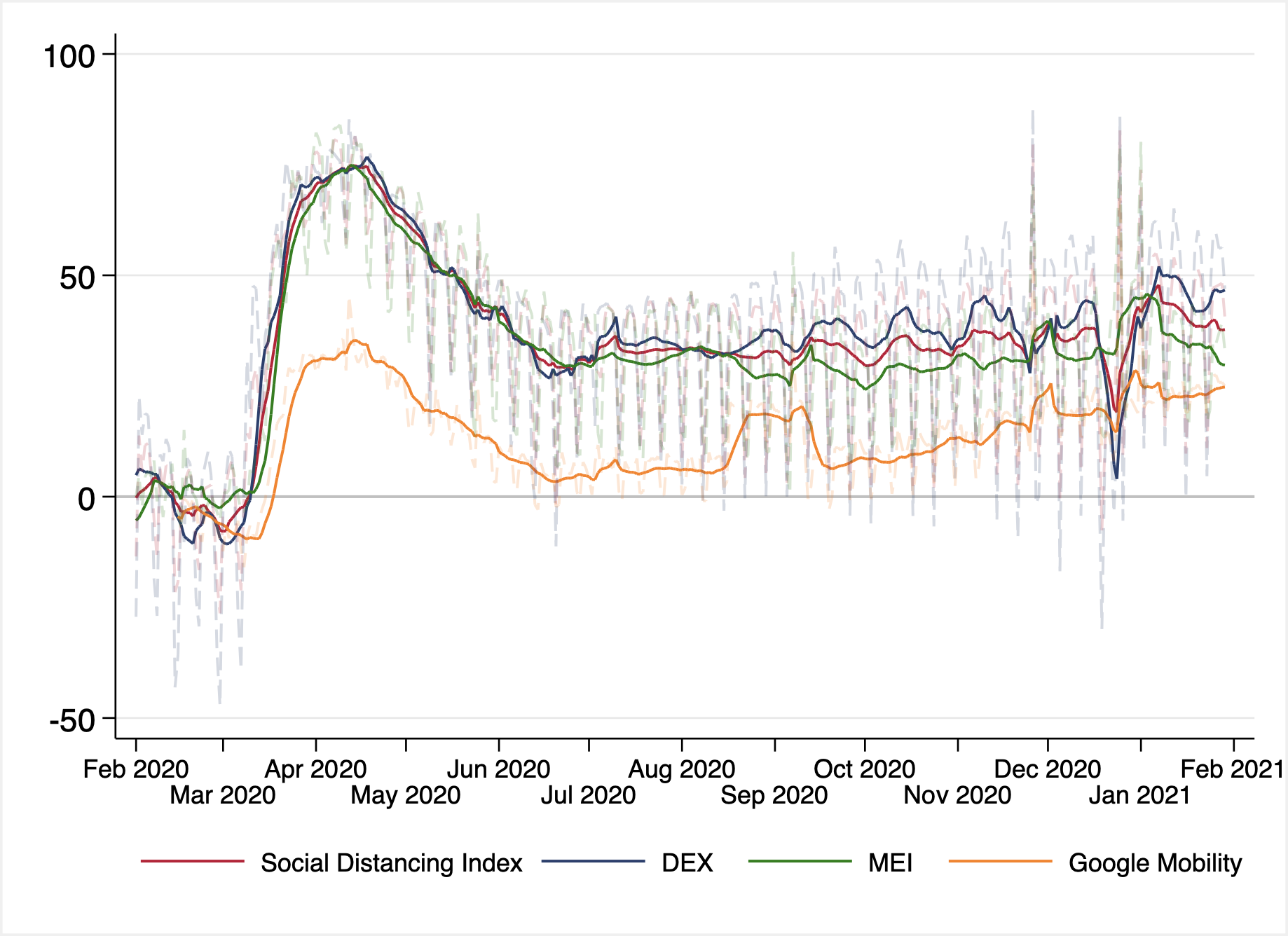

Supplement: S9 Data — (ZIP) [file pone.0267611.s010.zip › LaTeX/Fig/FigA3.2.png]

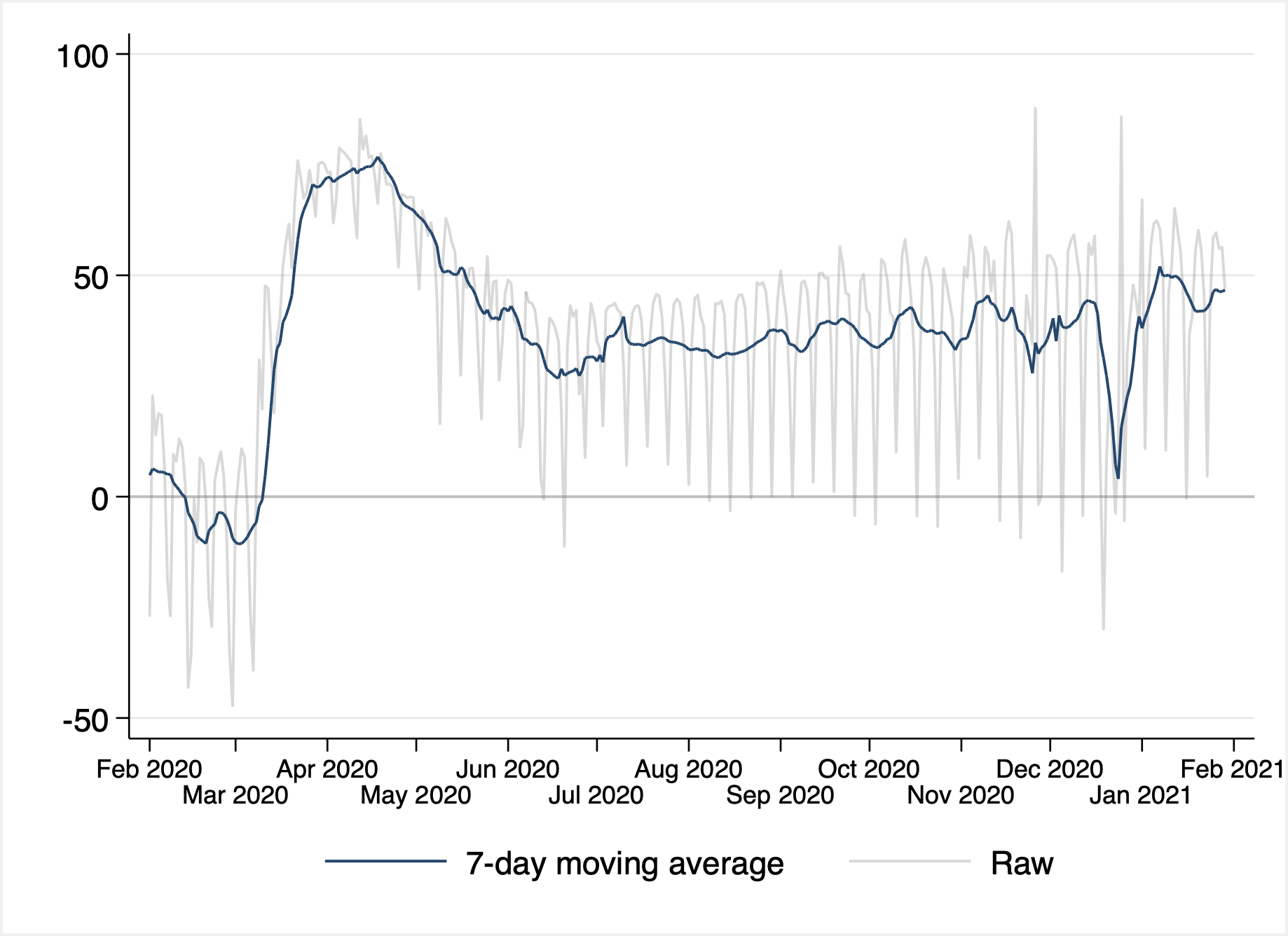

Supplement: S9 Data — (ZIP) [file pone.0267611.s010.zip › LaTeX/Fig/FigA3.1.png]
